# Enhanced Estrogenic Activity in Water-Soluble *Silybum marianum* Complex via Microwave-Assisted Steviol Glycoside-Based Formulation

**DOI:** 10.3390/nu18203388

**Published:** 2026-10-15

**Authors:** Ju-Hong Kim, Seong-Hun Jeong, Ba-Wool Lee, Ji-Hye Ha, Ju Hwan Jeong, Ji-Young Park, Hyung Jae Jeong, In-Chul Lee, Hyung-Jun Kwon, Kyuong-Oh Cho

**Affiliations:** 1 Functional Biomaterial Research Center, Korea Research Institute of Bioscience and Biotechnology (KRIBB), Jeongeup 56212, Republic of Korea; wnghd93@kribb.re.kr (J.-H.K.); jsh0830@kribb.re.kr (S.-H.J.); paul0706@kribb.re.kr (B.-W.L.); jihye2640@kribb.re.kr (J.-H.H.); juhwanjeong@kribb.re.kr (J.H.J.); loveme@kribb.re.kr (J.-Y.P.); hjeong21@kribb.re.kr (H.J.J.); leeic@kribb.re.kr (I.-C.L.); 2 Laboratory of Veterinary Pathology, College of Veterinary Medicine, Chonnam National University, Gwangju 61186, Republic of Korea

**Keywords:** estrogen receptor, microwave-assisted processing, phytoestrogen, *Silybum marianum*, steviol glycoside, water-soluble formulation

## Abstract

Background: Phytoestrogens are promising alternatives to menopausal hormone therapy, but their clinical application is limited by poor solubility and bioavailability. *Silybum marianum* (milk thistle) contains flavonolignans with reported estrogenic potential. However, its extract shows only modest estrogenic activity, and approaches to enhance its efficacy by improving solubility remain insufficiently explored. Methods: A water-soluble *Silybum marianum* complex (WS) was prepared using microwave-assisted processing with steviol glycoside (SG), and its phytochemical integrity was assessed by UPLC-QTOF-MS. Estrogenic activity was evaluated using cell-free ERα/ERβ competitive binding assays and, in MCF-7 cells, by assessing proliferation and ERα/ERK/AKT phosphorylation. In vivo efficacy was evaluated in a VCD-induced menopause model in female C57BL/6 mice by measuring plasma estradiol levels, uterine weight, and vaginal epithelial thickness. Results: WS retained major flavonolignans and incorporated SG-derived metabolites. WS bound both ERα and ERβ in cell-free assays. In MCF-7 cells, WS induced ERα, ERK, and AKT phosphorylation and cell proliferation; the proliferative response and ERK/AKT phosphorylation were abolished by the ER antagonist fulvestrant, and proliferation was comparable to that of genistein and daidzein. Neither *Silybum marianum* extract (SME) alone nor its simple physical combinations with SG reproduced the activity of WS, indicating that enhanced estrogenic activity was observed following microwave-assisted processing. In vivo, WS dose-dependently increased measured estradiol levels, increased uterine weight, and reversed vaginal atrophy. Conclusions: Microwave-assisted processing with SG significantly enhances the estrogenic activity of *Silybum marianum*. Our findings suggest that WS may serve as a promising phytoestrogenic candidate for further evaluation in the context of estrogen deficiency.

## 1. Introduction

As life expectancy increases globally, the proportion of older adults, particularly postmenopausal women, is rising rapidly. In Korea, life expectancy has markedly improved from 62.3 years among individuals born in 1970 to 82.7 years among those born in 2022. Notably, women have a higher life expectancy than men, living approximately 5.8 years longer on average [1]. Therefore, a substantial portion of women’s lives—often exceeding three decades—is now spent in the postmenopausal state [2].

Menopause, which typically occurs between the ages of 45 and 55, is characterized by ovarian follicular depletion, declining estrogen production, and elevated follicle-stimulating hormone (FSH) levels [3]. This hormonal transition leads to a broad spectrum of physical and psychological symptoms, including metabolic dysfunction, weight gain, increased cardiovascular risk, musculoskeletal changes, urogenital atrophy, and neuropsychiatric disturbances [4]. As postmenopausal life expectancy continues to increase, the need to understand and manage the long-term health consequences of estrogen deficiency becomes increasingly important. These considerations have led to increasing interest in menopausal hormone therapy (MHT) and alternative approaches to alleviating estrogen deficiency-related symptoms.

MHT, historically described as hormone replacement therapy, is widely employed to treat menopausal symptoms and prevent estrogen deficiency-related diseases such as osteoporosis and cardiovascular disorders. While MHT is clinically effective, its risk profile is not uniform: the risks of breast cancer, thrombosis, and stroke vary with the type and route of hormone used, the duration of treatment, and the age and time since menopause at initiation [5]. Concerns regarding the long-term safety of MHT have prompted many women to seek alternative therapies, particularly plant-derived extracts rich in phytoestrogens—bioactive compounds with estrogen-like properties that may help alleviate menopausal symptoms with a potentially more favorable safety profile [6].

Although phytoestrogens exhibit structural similarity to endogenous estrogens and can modulate estrogen receptor (ER) activity, their in vivo efficacy is often limited by poor water solubility, low bioavailability, and metabolic instability [7,8]. These factors hinder efficient absorption and target-tissue delivery, thereby reducing their functional potency in vivo [9]. Recent research has therefore focused on overcoming these limitations through formulation strategies that enhance solubility and systemic availability. Improving the pharmacokinetic profile of phytoestrogen-rich extracts may substantially enhance their therapeutic potential, particularly for managing postmenopausal symptoms and estrogen deficiency-related disorders [10,11,12].

*Silybum marianum*, widely recognized as milk thistle, is a member of the *Asteraceae* family and has long been used as a medicinal plant for treating liver disorders [13]. In addition to its hepatoprotective effects, it exhibits a wide spectrum of pharmacological activities, including neuroprotective, anticancer, antihypertensive, and hypolipidemic effects [14,15,16]. Among its bioactive constituents, silybin is recognized as the principal bioactive compound and is classified as a flavonolignan [17,18,19]. Silybin shares structural features with known phytoestrogens, and its capacity to interact with ERs has been reported in several in vitro studies, supporting its potential estrogenic activity [20,21]. However, the poor water solubility and physicochemical instability of *Silybum marianum* extract (SME) limit its absorption and biological efficacy [22].

In this study, we aimed to improve the aqueous dispersibility of SME and to evaluate its potential estrogenic activity using both in vitro (MCF-7 cells) and in vivo (4-vinylcyclohexene diepoxide [VCD]-induced menopausal mouse) models. The solubilization strategy was adapted from a recently reported microwave-assisted green method utilizing steviol glycosides (SG), which has been shown to enhance the aqueous solubility of phytochemicals [23]. We hypothesized that this approach would increase the functional availability of flavonolignans, thereby strengthening the estrogenic activity of SME.

## 2. Materials and Methods

### 2.1. Preparation of Water-Soluble Silybum marianum Complex (WS)

*Silybum marianum* extract (SME; NaturScent Co., Seoul, Republic of Korea) and steviol glycoside (SG; Macrocare, Cheongju, Republic of Korea) were used to formulate the water-soluble *Silybum marianum* complex (WS). For individual solutions, 100 mg of SME was dispersed in 5 mL of deionized water (DW), and 200 mg of SG was separately dissolved in 5 mL of DW. The SME dispersion and SG solution were combined to obtain a 10 mL mixture containing nominal concentrations of 10 mg/mL SME and 20 mg/mL SG, corresponding to an SME:SG mass ratio of 1:2. The mixture was subjected to microwave-assisted processing using a microwave reactor system (CEM, Matthews, NC, USA) at 150 W and 70 °C for 10 min. Following microwave treatment, the mixture was cooled, frozen, and lyophilized to obtain WS powder.

Prior to subsequent experiments, the entire lyophilized WS product prepared from 100 mg of SME and 200 mg of SG was dispersed in 2 mL of DW to prepare a stock dispersion with a nominal SME-equivalent loading of 50 mg/mL. This nominal value was calculated from the input mass of crude SME used during WS preparation. SME and SG constituents were qualitatively characterized by QTOF-MS. Individual silymarin constituents in SME were quantified by HPLC, whereas the principal constituents of SG, stevioside and rebaudioside A, were quantified by MS. However, experimental concentrations and doses were expressed as nominal SME-equivalent values and were not standardized to the measured contents of these constituents. Unless otherwise specified, in vitro concentrations are therefore expressed as nominal SME-equivalent values based on the initial mass of SME used for formulation. In the in vivo study, only WS was administered, and doses are reported as nominal SME-equivalent doses (mg/kg) calculated from the initial mass of SME incorporated during WS preparation.

### 2.2. UPLC–QTOF–MS Analysis

For qualitative profiling of WS and SME, we employed a Nexera XS UPLC instrument (Shimadzu, Kyoto, Japan) coupled to an X500R QTOF mass spectrometer (SCIEX, Framingham, MA, USA).

An ACQUITY UPLC BEH C18 column (2.1 × 100 mm, 1.7 μm; Waters, Milford, MA, USA) thermostated at 35 °C was used for chromatographic separation. Elution was performed with a binary mobile phase consisting of solvent A (0.1% formic acid in water) and solvent B (acetonitrile) under the following gradient conditions: 0 min, 95% A; 10 min, 75% A; 20 min, 50% A; 24 min, 50% A; 28 min, 10% A; 35 min, 10% A; and 45 min, 95% A. The flow rate was maintained at 0.3 mL/min, with an injection volume of 3 μL.

Mass spectrometric measurements were acquired in positive electrospray ionization (ESI^+^) mode using an information-dependent acquisition (IDA) strategy over an *m*/*z* range of 100–1500. The ion source temperature was set to 500 °C, with the curtain gas at 30 psi and ion source gases 1 and 2 adjusted to 50 psi. The ion spray voltage was +5500 V, and the declustering potential was fixed at +80 V. The collision energy was 35 ± 15 eV, and mass accuracy was kept within ±2 ppm during the runs.

Data were acquired and processed with SCIEX OS software (version 3.0.0.3339; SCIEX). Compounds were identified using accurate mass measurements, MS/MS fragmentation profiles, and comparison with reference databases.

### 2.3. HPLC Calibration Curves and Chromatographic Conditions

The quantitative determination of individual SME-derived flavonolignans and taxifolin in SME and WS was performed using values prepared from reference standards. Reference Standard solutions were prepared in methanol/water (35:65, *v*/*v*). The calibration ranges were 1–250 µg/mL for taxifolin, silydianin, and silybin B; 1–1200 µg/mL for silychristin; and 1–400 µg/mL for silybin A + isosilybin B and isosilybin A.

HPLC analysis was performed using an Agilent 1260 Infinity Series HPLC system (Agilent Technologies, Palo Alto, CA, USA) equipped with a degasser, binary pump, autosampler, diode-array detector (DAD), and column oven. Separation was achieved using a ZORBAX SB-C18 column (4.6 × 150 mm, 5 µm) maintained at 35 °C. The mobile phase consisted of 0.1% formic acid in water (solvent A) and 0.1% formic acid in methanol (solvent B). The gradient program was as follows: 0 min, 30% B; 32 min, 60% B; 35 min, 100% B; 38 min, 100% B; 38.5 min, 30% B; and 46 min, 30% B. The flow rate was 1.0 mL/min, and the injection volume was 10 µL.

Calibration curves were constructed by plotting the peak area against the corresponding standard concentration. The regression equations for taxifolin, silychristin, silydianin, silybin B, silybin A + isosilybin B, and isosilybin A were y = 32.144x + 4.071 (R^2^ = 0.9999), y = 17.512x + 136.21 (R^2^ = 0.9972), y = 15.321x − 0.7793 (R^2^ = 1.0000), y = 11.25x − 4.8443 (R^2^ = 0.9999), y = 47.232x + 122.36 (R^2^ = 0.9993), and y = 11.989x + 13.667 (R^2^ = 0.9969), respectively.

For sample preparation, the WS stock dispersion (50 mg/mL nominal SME equivalent), prepared as described above, was diluted 50-fold with DW to obtain a nominal SME-equivalent concentration of 1 mg/mL. SME was separately dispersed in DW at the same nominal SME-equivalent concentration. The WS and SME dispersions were thoroughly mixed by vortexing. The dispersions were subsequently filtered through a 0.20-µm hydrophilic PTFE syringe filter (13 mm, ADVANTEC, Tokyo, Japan). The filtrates were transferred to HPLC vials and analyzed under the chromatographic conditions described above. Quantification of each component was performed using the corresponding external calibration curve.

### 2.4. LC–MS/MS Calibration and Quantification

WS concentrations are expressed on a nominal SME-equivalent basis. The WS stock dispersion was prepared with SME and SG at nominal concentrations of 50 and 100 mg/mL, respectively, corresponding to a total nominal WS loading of 3 mg/mL. This stock dispersion was diluted 50-fold with distilled water (DW) to obtain a nominal SME-equivalent concentration of 1 mg/mL (nominal SG concentration, 2 mg/mL; total nominal WS loading, 3 mg/mL), followed by filtration through a 0.20-µm hydrophilic PTFE syringe filter (13 mm, ADVANTEC, Japan). The resulting filtrate was further diluted 100-fold with DW, followed by an additional 100-fold dilution (overall 10,000-fold dilution of the filtrate) prior to LC–MS/MS analysis. Concentrations of stevioside and rebaudioside A are reported relative to the 0.20-µm filtrate.

Stevioside and rebaudioside A in WS were quantified using an Agilent 1260 HPLC system (Agilent Technologies, USA) coupled to a SCIEX API 4000 triple quadrupole mass spectrometer (SCIEX, USA). Calibration standards were prepared at concentrations of 1, 5, 10, 50, 100, 500, and 1000 ng/mL, covering a range of 1–1000 ng/mL. Chromatographic separation was performed using a C18-AQ column (2.1 × 100 mm, 3 µm) maintained at 35 °C. The mobile phases consisted of 0.1% formic acid in water (A) and 0.1% formic acid in acetonitrile (B). The gradient was initiated at 10% B, increased to 95% B over 12 min, and maintained at 95% B for an additional 15 min.

Mass spectrometric detection was performed using an electrospray ionization source in negative-ion multiple-reaction-monitoring (MRM) mode. The optimized MRM transitions and mass spectrometric parameters for stevioside and rebaudioside A are provided in Table 1. WS samples were diluted 100-fold with distilled water and then subjected to a further 100-fold dilution before LC–MS/MS analysis, yielding an overall dilution factor of 10,000. Calibration curves were generated by plotting the analytical response against the corresponding standard concentration. The regression equations for rebaudioside A and stevioside were y = 2.85 × 10^3^x + 1.57 × 10^4^ (r = 0.9991) and y = 1.99 × 10^3^x + 1.31 × 10^4^ (r = 0.9989), respectively.

### 2.5. Cell Culture and E-Screen Assay

The ER-positive MCF-7 human breast cancer cell line (ATCC, Manassas, VA, USA) was cultured in RPMI-1640 medium (Gibco, Grand Island, NY, USA) supplemented with 10% fetal bovine serum (FBS; Gibco, Grand Island, NY, USA), 100 U/mL penicillin, and 100 μg/mL streptomycin. Cells were maintained at 37 °C in a humidified incubator with 5% CO_2_ and 95% air.

To evaluate estrogenic activity, MCF-7 cells were seeded in 96-well plates at a density of 5 × 10^3^ cells per well in phenol red-free RPMI-1640 medium (Gibco, Grand Island, NY, USA) supplemented with 10% FBS, 100 U/mL penicillin, and 100 μg/mL streptomycin. After 24 h, the medium was replaced with phenol red-free RPMI-1640 containing 5% charcoal-dextran–stripped FBS (CD-FBS).

Cells were then treated with SME, microwave-treated SME (SME + M), the physical mixture of SME and SG (SME + SG), or WS at matched nominal SME-equivalent concentrations of 10, 25, and 50 μg/mL. For cell treatment, the WS stock dispersion (50 mg/mL nominal SME equivalent) and the other test materials were diluted in the culture medium to achieve the indicated final concentrations. SME + M was prepared by subjecting SME to the same microwave treatment used for WS preparation, but without SG. SME + SG was freshly prepared by physically mixing SME and SG at a 1:2 mass ratio without microwave-assisted processing or freeze-drying, whereas WS was prepared as described in Section 2.1. SG was tested at nominal final concentrations of 10, 25, and 50 μg/mL based on SG mass. Estradiol (E2; 10^−8^ M; Sigma-Aldrich, St. Louis, MO, USA) was used as a positive control. Individual SME-derived flavonolignans and taxifolin, including silybin A, silybin B, silychristin, silydianin, isosilybin A, and isosilybin B (all from Phytolab, Vestenbergsgreuth, Germany), as well as taxifolin (Tokyo Chemical Industry, Tokyo, Japan), were also evaluated at the indicated concentrations.

Treatments were conducted in the presence or absence of the ER antagonist fulvestrant (10^−6^ M; Sigma-Aldrich, St. Louis, MO, USA). After 144 h of incubation, cell proliferation was assessed using the MTT assay. MTT reagent was added and incubated for 3–4 h, and absorbance was measured at 570 nm using a microplate reader (SpectraMax M2e, Molecular Devices, Sunnyvale, CA, USA) in an MCF-7 estrogen-dependent proliferation assay adapted from previously described protocols, with minor modifications [24].

### 2.6. Additional Proliferation Assays

To complement the MTT assay and further validate the effects of treatments on cell proliferation, two additional assays were conducted: a 5-bromo-2′-deoxyuridine (BrdU) incorporation assay and trypan blue dye exclusion staining.

For the BrdU incorporation assay, MCF-7 cells were seeded in 96-well plates and treated under the same conditions described for the E-screen assay. After the designated treatment period, cell proliferation was assessed using a BrdU Cell Proliferation ELISA Kit (Abcam, Cambridge, UK), following the manufacturer’s instructions.

For trypan blue staining, cells were harvested after treatment and mixed at a 1:1 ratio with 0.4% trypan blue solution (Gibco, Grand Island, NY, USA). Viable (unstained) and non-viable (stained) cells were counted using an automated cell counter, and total cell numbers and cell viability were determined.

### 2.7. ER Binding Assay

The binding affinity of WS to ER was assessed using the PolarScreen™ ER Competitor Assay Kit (Thermo Fisher Scientific, Waltham, MA, USA), according to the manufacturer’s instructions. Separate assays were performed for ERα and ERβ.

Fluorescence polarization was measured using a microplate reader (Molecular Devices, Sunnyvale, CA, USA). In this competitive binding assay, a high polarization value (MAX) indicates the formation of a stable complex between the nuclear receptor (ERα or ERβ) and the Fluormone™ fluorescent tracer (Thermo Fisher Scientific). In contrast, a low polarization value (MIN) occurs when the tracer is displaced by a competing ligand such as E2, indicating receptor-binding activity of the test compound.

The binding activity of WS was calculated as percent inhibition using the following formula:

Inhibition (%) = 100 × [(X − MAX)/(MIN − MAX)],

where X represents the polarization value following WS treatment. A higher percent inhibition reflects a stronger binding affinity of WS to the ER, as it effectively competes with the tracer for receptor binding.

### 2.8. Western Blot Analysis

MCF-7 cells were seeded in 6-well plates at a density of 2 × 10^5^ cells per well in phenol red-free RPMI-1640 medium, supplemented with 10% FBS, 100 U/mL penicillin, and 100 μg/mL streptomycin. After 24 h of incubation at 37 °C in a humidified 5% CO_2_ atmosphere, the medium was replaced with phenol red-free RPMI-1640 containing 5% charcoal-dextran–stripped FBS (CD-FBS).

Western blot analysis was conducted following a previously described protocol [25]. The primary antibodies used in this study were as follows: ERα (Cat. No. 8644), phospho-ERα (*p*-ERα; Cat. No. 2511), ERK (Cat. No. 9102), phospho-ERK (*p*-ERK; Cat. No. 9101), AKT (Cat. No. 9272), and phospho-AKT (*p*-AKT; Cat. No. 4060) (1:1000 dilution, Cell Signaling Technology, Danvers, MA, USA). β-actin (Cat. No. PA1-183; Thermo Fisher Scientific, Waltham, MA, USA) was used as a loading control. Chemiluminescent signals were captured using a chemiluminescent imaging scanner (LI-COR Biosciences, Lincoln, NE, USA), and protein band intensities were quantified using ImageJ software 1.54g (National Institutes of Health, Bethesda, MD, USA). All Western blot experiments were performed using three independent biological replicates (*n* = 3), each using a separate cell passage and a separate membrane, and phosphorylated protein levels were normalized to the corresponding total protein level for each target on the same membrane.

### 2.9. Animal Preparation, Experimental Design, and VCD Administration

Female C57BL/6 mice (6–8 weeks old, 25–28 g) were purchased from Orient Bio Inc. (Seongnam, Gyeonggi-do, Republic of Korea) and acclimated for one week under standard laboratory conditions (23 ± 3 °C, 50 ± 10% humidity, 12 h light/dark cycle) with ad libitum access to food and water. All animal experiments were approved by the Institutional Animal Care and Use Committee (approval number: KRIBB-AEC-23114).

To induce menopause, mice were intraperitoneally injected with VCD (160 mg/kg/day, dissolved in corn oil) for 20 consecutive days. Menopausal status was confirmed by persistent diestrus over 10 days using vaginal smear cytology [26].

Following confirmation of the menopausal status, animals were randomly divided into six experimental groups, each consisting of eight mice. One group served as the normal control and received no treatment. A second group, representing the menopausal control, received VCD only. Another group was treated with E2 at a dose of 50 μg/kg, administered subcutaneously three times per week, serving as the positive control. The remaining three groups received WS extract orally at doses of 10, 50, or 100 mg/kg/day for a total of 10 weeks.

**Figure 1:**
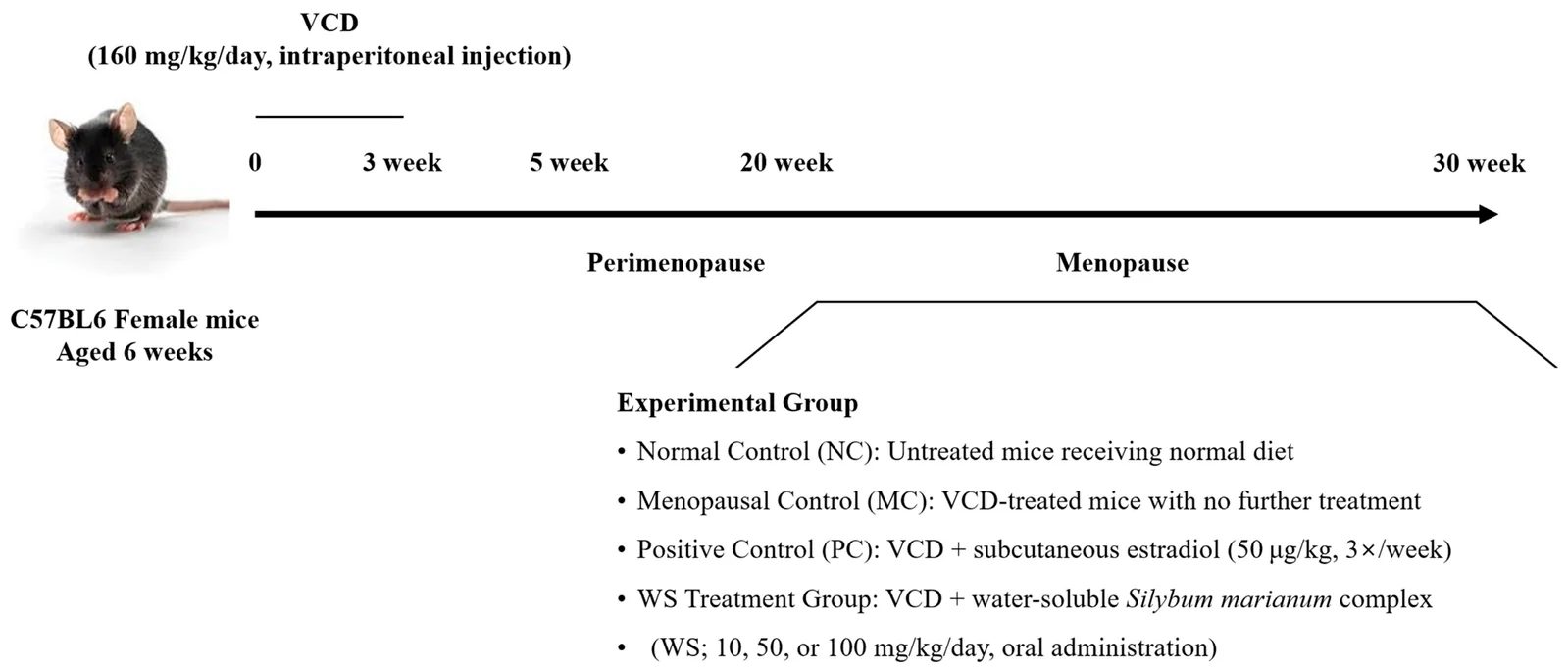
Schematic representation of the in vivo experimental design. Female C57BL/6 mice were administered 4-vinylcyclohexene diepoxide (VCD; 160 mg/kg/day, intraperitoneally) for 20 consecutive days to induce menopause, except for mice assigned to the normal control (NC) group, which did not receive VCD. Following confirmation of persistent diestrus in VCD-treated mice, the animals were randomly assigned to the menopausal control (MC), positive control (PC), and WS treatment groups. The NC group received neither VCD nor subsequent treatment. The MC group received VCD only. The PC group received VCD followed by subcutaneous administration of estradiol (E2; 50 μg/kg) three times per week. The remaining VCD-treated mice were assigned to WS treatment groups and received daily oral administration of water-soluble *Silybum marianum* complex (WS) at doses of 10, 50, or 100 mg/kg/day for 10 weeks. Tissues and blood samples were collected at the end of the study.

An overview of the experimental timeline and treatment assignments is illustrated in Figure 1. At the end of the study, animals were sacrificed, and biological samples including blood, uterine, and vaginal tissues were collected for further analysis.

### 2.10. Measurement of Plasma Estradiol Levels

Blood samples were collected from the inferior vena cava under anesthesia at the time of sacrifice. Plasma was separated by centrifugation at 6000× *g* for 10 min. Estradiol concentrations were determined using a commercially available ELISA kit (Cat. No. MBS701244; MyBioSource, San Diego, CA, USA) according to the manufacturer’s instructions.

### 2.11. Histopathological Analysis

The vaginal tissue samples were rinsed with saline to remove residual blood and fixed in 10% phosphate-buffered formalin for 48 h. Following fixation, tissues were embedded in paraffin and sectioned into 4 μm slices. The tissue sections were stained with hematoxylin and eosin (H&E; BBC Biochemical, Mount Vernon, WA, USA) according to standard histological protocols. The stained samples were then examined under a light microscope (Leica Microsystems, Wetzlar, Germany) to assess vaginal epithelial thickness [27]. The observer was blinded to treatment group assignment during image acquisition and quantification. The vaginal epithelial thickness was quantified using ImageJ software (NIH, Bethesda, MD, USA).

### 2.12. Statistical Analysis

All data are presented as means ± standard deviations (SDs). Statistical analyses were performed using GraphPad Prism software (version 5.0; GraphPad Software Inc., San Diego, CA, USA). For in vitro experiments, *n* refers to the number of independent experiments; technical replicates within each experiment were averaged to a single value before analysis. For in vivo experiments, *n* refers to the number of animals per group. For both in vitro and in vivo experiments involving more than two groups, the nonparametric Kruskal–Wallis test was applied, given the small sample sizes and the potential violations of normality assumptions. When significant differences were observed, Dunn’s multiple comparisons test was used for post hoc pairwise comparisons. A *p* < 0.05 was considered statistically significant, and *p* < 0.001 was considered highly significant.

## 3. Results

### 3.1. Water-Soluble Silybum marianum Complex (WS) Enhances Estrogenic Activity in MCF-7 Cells

**Figure 2:**
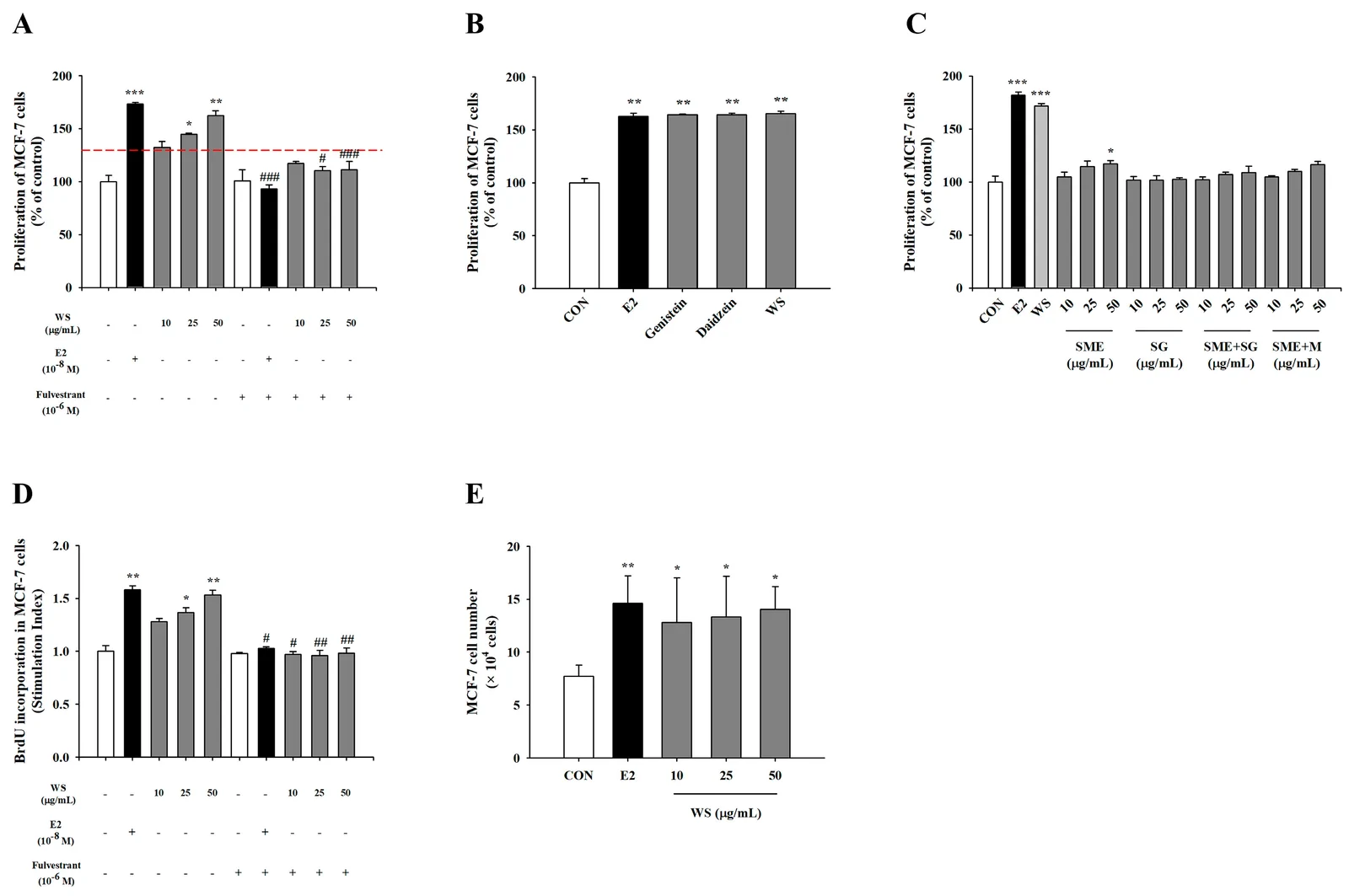
WS promotes estrogen receptor-dependent proliferation in MCF-7 cells. (**A**) MCF-7 cell proliferation assessed using the E-screen assay and expressed as a percentage of the untreated control, which was set to 100%. Cells were treated with water-soluble *silybum marianum* complex (WS) in the absence or presence of fulvestrant (10^−6^ M) to assess estrogen receptor dependence. The red dashed line indicates the 130% threshold used in this study to classify estrogenic activity. (**B**) Comparison of WS-induced proliferation with that induced by the phytoestrogens genistein (10^−6^ M) and daidzein (10^−5^ M). (**C**) Comparative effects of *Silybum marianum* extract (SME), steviol glycoside (SG), physical mixture of SME and SG (SME + SG), and microwave-treated SME (SME + M) on MCF-7 cell proliferation. (**D**) DNA synthesis assessed by BrdU incorporation assay. (**E**) Viable cell number quantified by trypan blue exclusion. In panels (**C**–**E**), CON denotes untreated control cells. Data are presented as means ± SDs (*n* = 6 independent experiments). Statistical significance was determined using the Kruskal–Wallis nonparametric test followed by Dunn’s multiple comparison test. * *p* < 0.05, ** *p* < 0.01, and *** *p* < 0.001 vs. untreated control cells; # *p* < 0.05, ## *p* < 0.01, and ### *p* < 0.001 vs. corresponding treatment without fulvestrant.

To evaluate the estrogenic activity of the water-soluble *Silybum marianum* complex (WS), we first examined its effects on estrogen receptor (ER)-positive MCF-7 cell proliferation. We also compared WS with the *Silybum marianum* extract (SME) and steviol glycoside (SG), alone and in combination, to determine whether microwave-assisted processing with SG contributes to the observed activity. Estradiol (E2, 10^−8^ M) significantly increased cell proliferation to 173.19% of control levels, and this response was abolished in the presence of the ER antagonist fulvestrant, indicating ER-mediated action. WS treatment also induced a dose-dependent increase in cell proliferation, reaching 132.35%, 144.83%, and 162.27% at 10, 25, and 50 μg/mL, respectively. These proliferative effects were similarly reversed by fulvestrant, indicating involvement of ER signaling (Figure 2A).

BrdU incorporation assays showed corresponding increases in DNA synthesis (1.28–1.53-fold), comparable to E2 (1.58-fold), and trypan blue exclusion confirmed increased viable cell numbers across all WS concentrations (Figure 2D,E). WS-induced proliferation was also compared with that induced by the phytoestrogens genistein and daidzein, and the extent of proliferation was comparable among the compounds, suggesting that WS exerts phytoestrogen-like activity (Figure 2B).

To determine whether this effect was dependent on microwave-assisted processing, comparisons were made between WS, SME, SG, their physical mixture of SME and SG (SME + SG), and microwave-treated SME (SME + M). Only WS induced strong proliferation, whereas other formulations showed minimal or no effect (Figure 2C), indicating that the estrogenic activity is specific to the WS formulation.

### 3.2. Chemical Characterization and Aqueous Filtration Analysis of SME and WS

#### 3.2.1. UPLC-QTOF-MS Chemical Profiling

**Figure 3:**
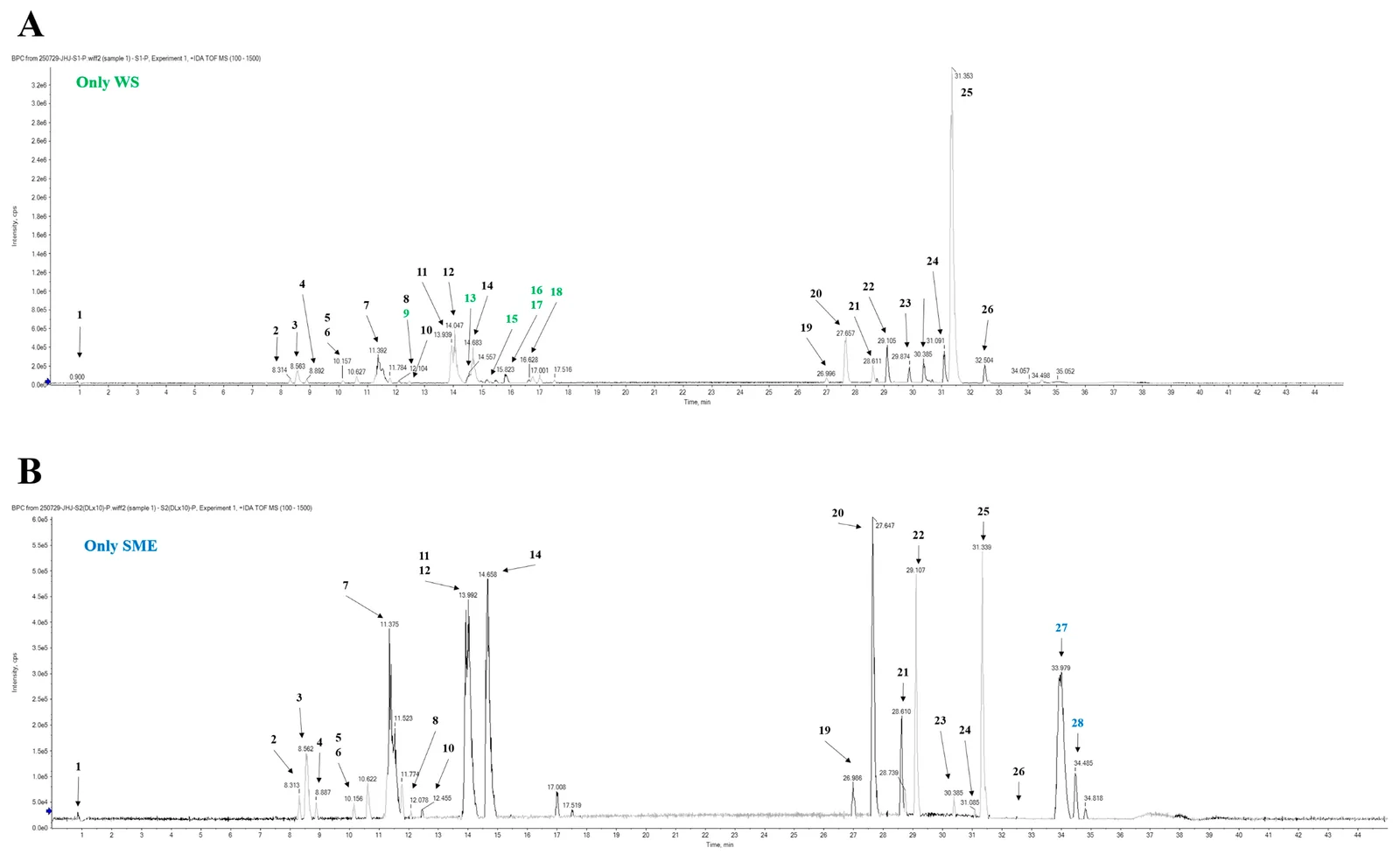
UPLC-ESI-QTOF-MS base peak chromatograms (BPCs) of *Silybum marianum* acquired in positive ion mode. (**A**) Water-soluble *Silybum marianum* complex (WS); (**B**) *Silybum marianum* extract (SME). Peaks common to both samples are indicated in black; WS-specific peaks are shown in green, and SME-specific peaks are shown in blue.

UPLC–QTOF–MS/MS profiling revealed that both WS and SME retained the major flavonolignans of *Silybum marianum*, including silybin, isosilybin, and silychristin, along with flavonoids such as quercetin and naringenin (Figure 3, Table 2) [28]. These compounds were putatively identified based on accurate mass and MS/MS fragmentation patterns. These findings indicate that the microwave-assisted processing preserved the primary phytochemical constituents of the extract.

In addition, several SG-derived metabolites—including steviol, isosteviol, and 6α-mannobiose—were uniquely putatively identified in WS, suggesting compositional changes introduced by SG incorporation [29].

Collectively, these results demonstrate that WS retains the key flavonolignans of *Silybum marianum* while introducing putatively identified SG-derived metabolites through microwave-assisted processing. This compositional shift may underlie the enhanced estrogenic activity observed in subsequent assays.

#### 3.2.2. Quantitative Characterization of WS

The quantified SME- and SG-derived analytes in the 0.20-µm-filterable fraction were evaluated relative to the total nominal WS loading initially introduced (3 mg/mL, comprising 1 mg/mL SME and 2 mg/mL SG) (Table 3). The SME-derived flavonolignans and taxifolin (229.16 ± 3.35 µg/mL) accounted for approximately 7.6% of the total nominal WS loading, while the combined stevioside and rebaudioside A (1.271 ± 0.025 mg/mL) accounted for approximately 42.4%. The remaining fraction reflects both non-targeted minor constituents inherent to the crude SME and SG extracts and any material retained by the 0.20-µm membrane filter during sample preparation, rather than analytical loss during HPLC or LC–MS/MS quantification.

#### 3.2.3. Aqueous Dispersion and Filtration Analysis

**Figure 4:**
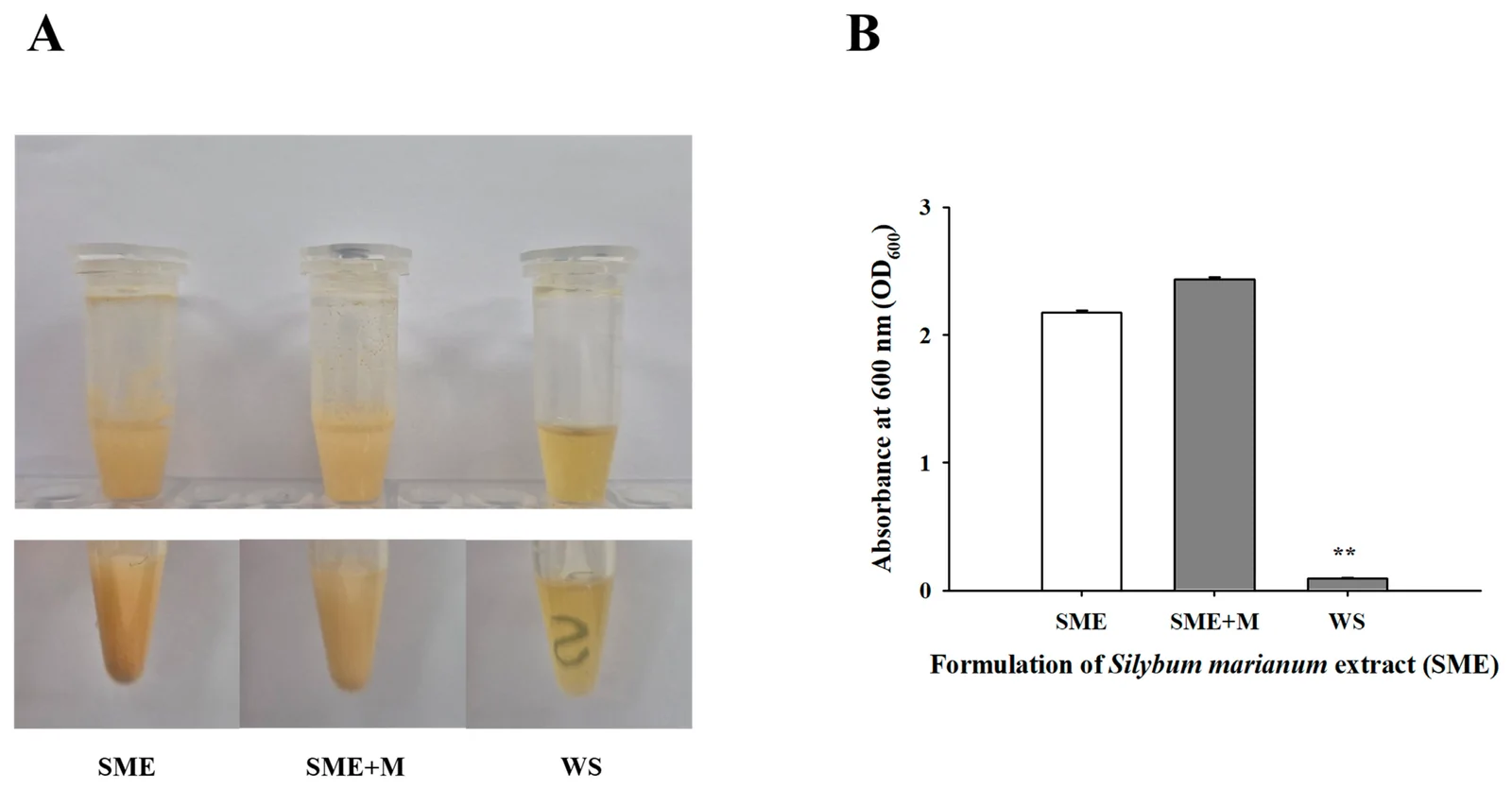
Visual appearance and turbidity of *Silybum marianum* formulations in DW. (**A**) Representative images of *Silybum marianum* extract (SME), microwave-treated SME (SME + M), and water-soluble *Silybum marianum* complex (WS) dispersed in deionized water (DW) at a nominal SME-equivalent loading of 50 mg/mL. The letter “S” positioned behind the tubes was used as a visual indicator of turbidity; it was visible through the WS sample but obscured by the SME + M and SME, indicating lower turbidity of WS. (**B**) Optical density at 600 nm (OD_600_) of each formulation measured in a microplate reader (*n* = 3 independent experiments). Statistical significance was determined using the Kruskal–Wallis nonparametric test followed by Dunn’s multiple comparison test. ** *p* < 0.01 vs. SME.

The aqueous dispersion characteristics of SME, SME + M, and WS were evaluated in DW at nominal SME-equivalent loading of 50 mg/mL. As shown in Figure 4A, the letter positioned behind the sample tubes was visible through the WS dispersion but was obscured by the SME and SME + M dispersions. In addition, WS showed a lower OD_600_ than SME (Figure 4B).

The concentrations of individual SME-derived flavonolignans and taxifolin in the 0.20-µm-filterable aqueous fractions were subsequently determined by HPLC. SME and WS were dispersed in deionized water at a nominal SME-equivalent loading of 1 mg/mL and filtered through a 0.20-µm hydrophilic PTFE syringe filter before analysis. As summarized in Table 4, silybin A + isosilybin B, silybin B, and isosilybin A were not detected in the SME filtrate but were quantified in the WS filtrate. The total concentration of quantified SME-derived flavonolignans and taxifolin was 15.37 ± 0.11 µg/mL for SME and 231.48 ± 2.00 µg/mL for WS.

### 3.3. WS Binds to Nuclear Estrogen Receptors ERα and ERβ

**Figure 5:**
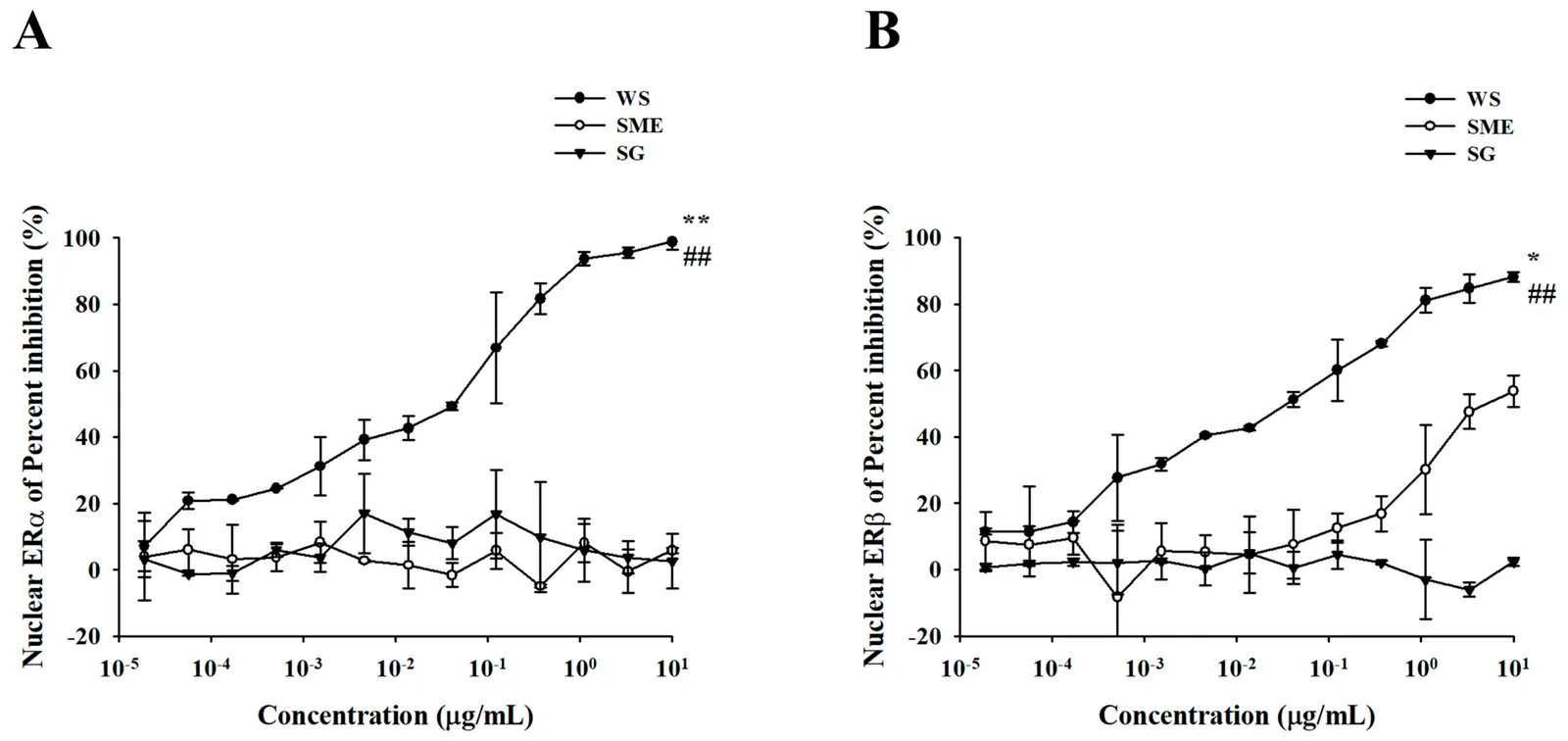
WS competitively binds to the nuclear estrogen receptor. (**A**) Competitive binding assay showing the inhibition of ligand binding to nuclear estrogen receptor α (ERα) by water-soluble *Silybum marianum* complex (WS), *Silybum marianum* extract (SME), and steviol glycoside (SG) at the indicated concentrations. (**B**) Competitive binding assay showing inhibition of ligand binding to nuclear estrogen receptor β (ERβ) under the same experimental conditions. Percent inhibition was calculated relative to reference ligand binding. Data are presented as means ± SDs (*n* = 3 independent experiments). Statistical significance was determined using the Kruskal–Wallis nonparametric test followed by Dunn’s multiple comparison test. * *p* < 0.05 and ** *p* < 0.01 vs. SME; ## *p* < 0.01 vs. SG.

To elucidate the mechanistic basis of the estrogenic activity observed with WS, its binding affinity to nuclear ERα and ERβ was assessed using a fluorescence polarization-based competitive binding assay. WS demonstrated a clear concentration-dependent increase in percent inhibition for both ERα (Figure 5A) and ERβ (Figure 5B), indicative of effective receptor binding. In contrast, SME and SG showed negligible binding activity across all concentrations. SME induced only a minimal response toward ERβ at high concentrations tested; its interaction with ERα remained marginal [20,21]. SG showed no measurable interaction with either receptor subtype. Notably, WS achieved over 80–90% inhibition at the highest concentration tested, reflecting a strong capacity to competitively displace the fluorescent tracer bound to both ER subtypes.

These findings show that, unlike SME and SG individually, WS binds effectively to both ERα and ERβ, consistent with its potential contribution to the estrogenic activity observed in cellular assays.

### 3.4. WS Activated ER Signaling Pathway in MCF-7 Cells

**Figure 6:**
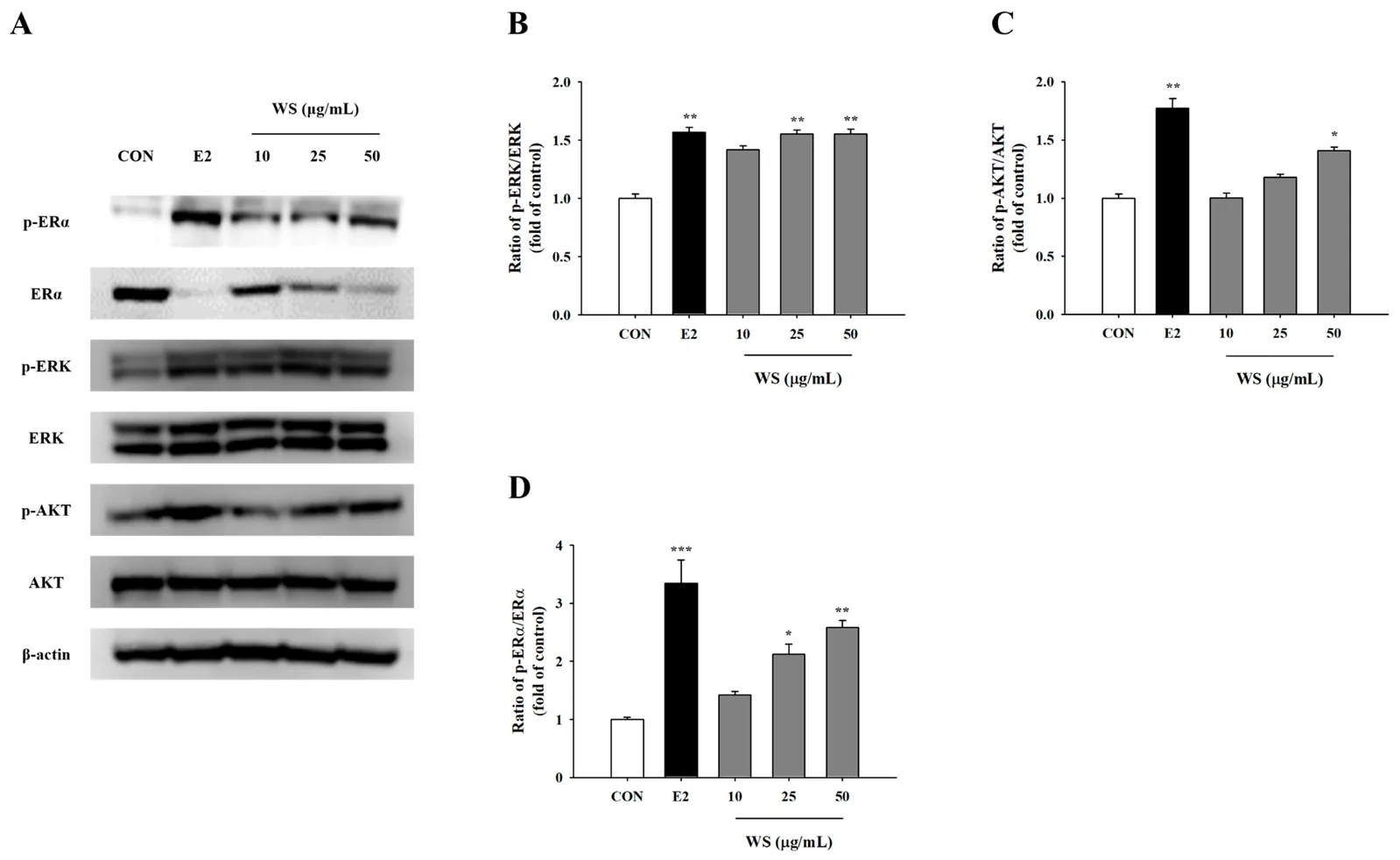
WS activates ER-mediated intracellular signaling pathways in MCF-7 cells. (**A**) Representative Western blot images showing the expression of phosphorylated ERK (p-ERK), ERK, phosphorylated AKT (p-AKT), AKT, phosphorylated ERα (p-ERα), ERα, and β-actin in MCF-7 cells treated with estradiol (E2; 10^−8^ M) or water-soluble *Silybum marianum* complex (WS; 10, 25, or 50 μg/mL). (**B**–**D**) Densitometric quantification of phosphorylation ratios: (**B**) *p*-ERK/ERK, (**C**) *p*-AKT/AKT, and (**D**) *p*-ERα/ERα. Phosphorylation ratios were normalized to the untreated control (CON), which was set to 1. Data are presented as means ± SDs (*n* = 3 independent experiments). Statistical analysis was performed using the Kruskal–Wallis nonparametric test followed by Dunn’s multiple comparison test. * *p* < 0.05, ** *p* < 0.01, and *** *p* < 0.001 vs. CON.

To further elucidate the mechanism underlying the estrogenic activity of WS, its effects on ER-mediated intracellular signaling pathways were examined in MCF-7 cells using Western blot analysis [30]. Treatment with E2 (10^−8^ M) significantly increased phosphorylation of AKT, ERK, and ERα, consistent with activation of classical ER signaling. Similarly, WS treatment induced a dose-dependent increase in phosphorylation levels of AKT, ERK, and ERα at 10, 25, and 50 μg/mL (Figure 6).

**Figure 7:**
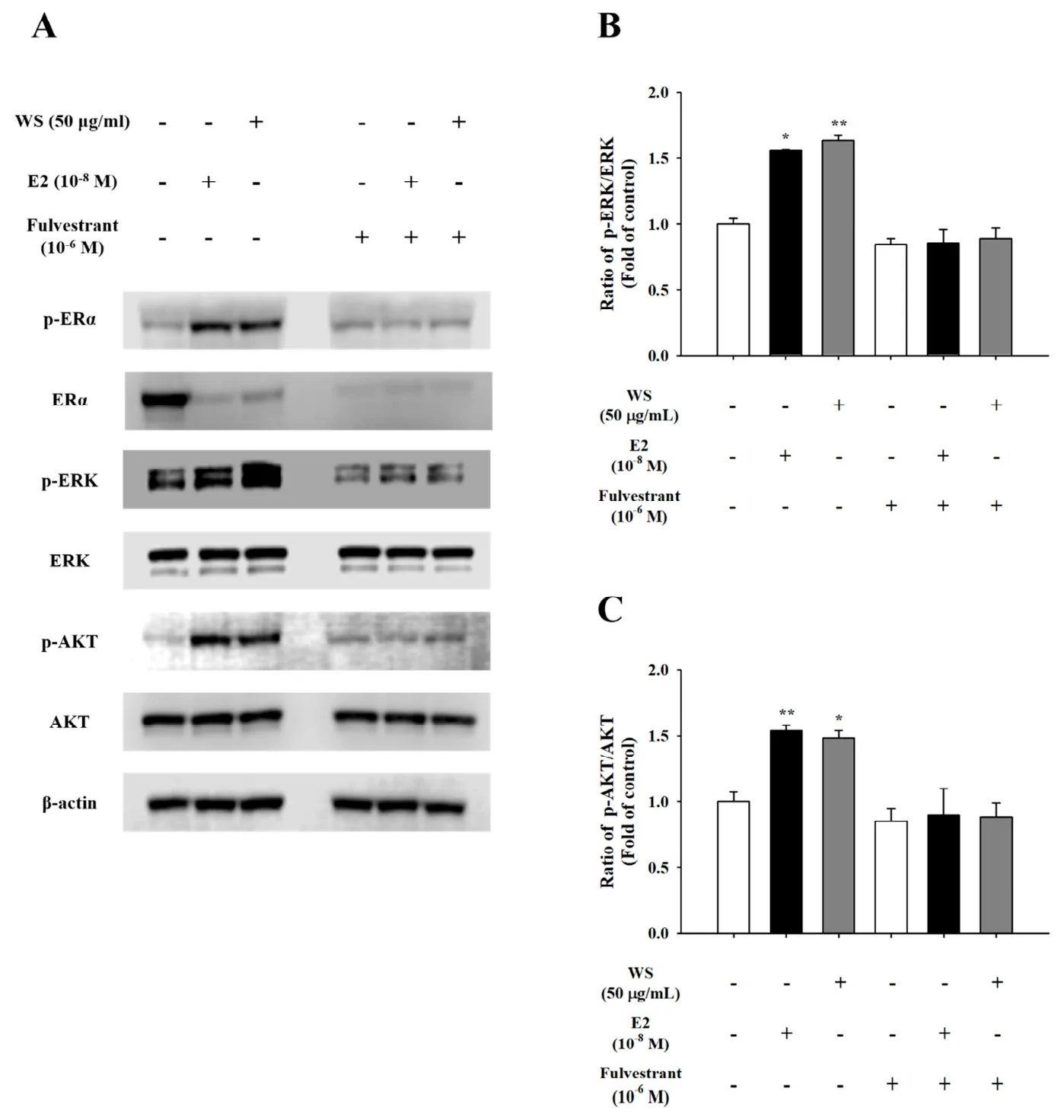
WS activates ER-mediated signaling in MCF-7 cells, and this activation is abolished by fulvestrant. (**A**) Representative Western blot images showing the expression of phosphorylated ERK (p-ERK), ERK, phosphorylated AKT (p-AKT), AKT, ERα, and β-actin in MCF-7 cells treated with water-soluble *Silybum marianum* complex (WS; 50 μg/mL), estradiol (E2; 10^−8^ M), and/or the ER antagonist fulvestrant (10^−6^ M). (**B**,**C**) Densitometric quantification of phosphorylation ratios: (**B**) *p*-ERK/ERK and (**C**) *p*-AKT/AKT. Phosphorylation ratios were normalized to the untreated control, which was set to 1. Data are presented as means ± SDs (*n* = 3 independent experiments). Statistical analysis was performed using the Kruskal–Wallis nonparametric test followed by Dunn’s multiple comparison test. * *p* < 0.05 and ** *p* < 0.01 vs. corresponding treatment with fulvestrant.

To confirm ER dependency, co-treatment with the ER antagonist fulvestrant (10^−6^ M) was performed. Fulvestrant markedly reduced WS- and E2-induced phosphorylation of AKT and ERK, supporting the involvement of an ER-mediated mechanism (Figure 7). Furthermore, both total and phosphorylated ERα levels were decreased upon fulvestrant treatment, consistent with its dual action as an ER antagonist and degrader via the proteasome pathway [31].

These findings demonstrate that WS activates intracellular estrogen signaling pathways in a dose-dependent and ER-mediated manner, resembling the effects of physiological estrogen.

### 3.5. Effects of WS in VCD-Induced Menopausal Mice

To evaluate the in vivo estrogenic effects of WS, a well-established VCD-induced menopausal mouse model was employed. Plasma E2 levels, uterine weight, and vaginal epithelial morphology were assessed as key indicators of estrogenic response [26].

**Figure 8:**
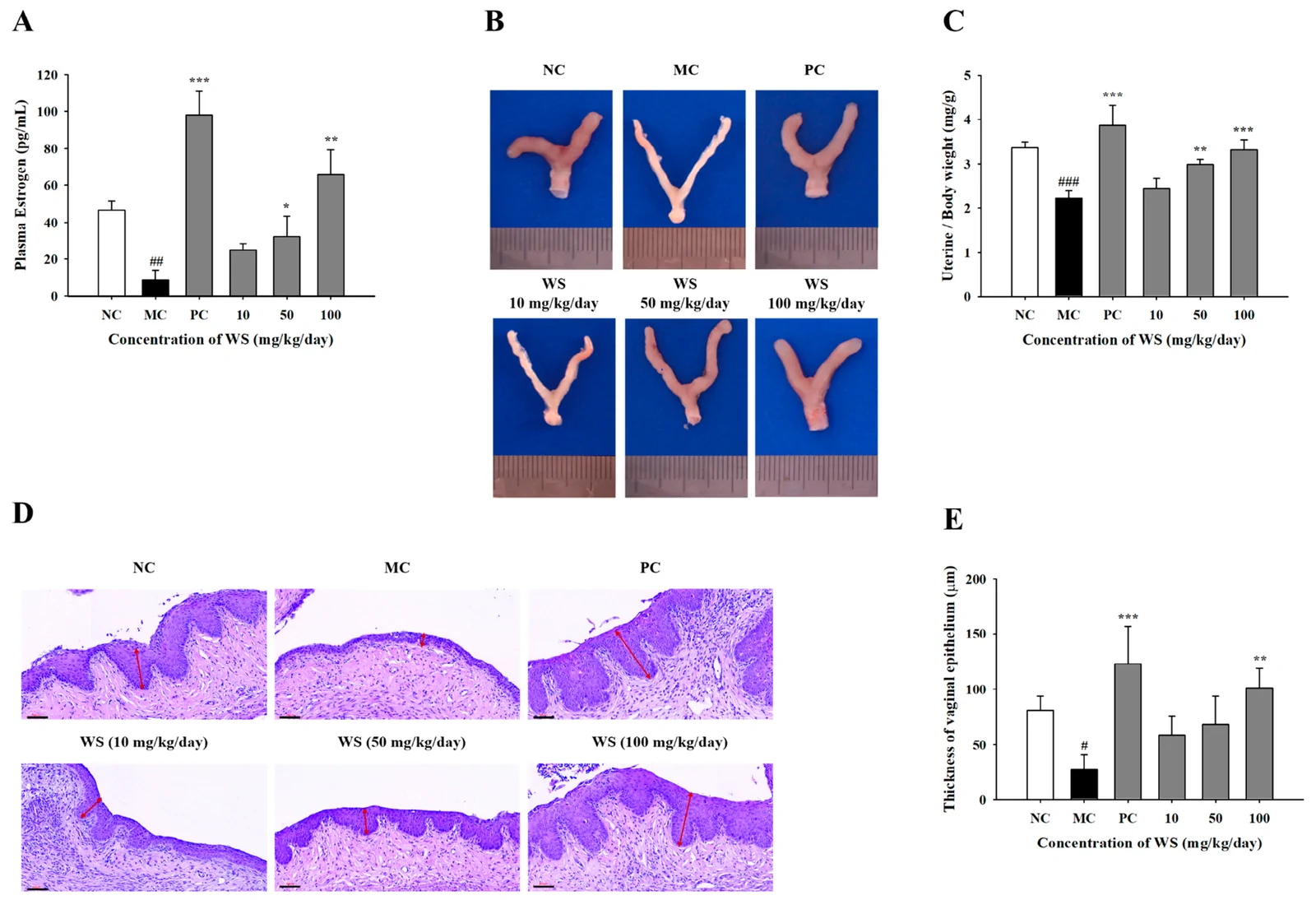
WS treatment altered plasma E2 concentration, relative uterine weight, and vaginal epithelial thickness in VCD-induced menopausal mice. (**A**) Plasma estradiol (E2) concentrations measured by ELISA. (**B**) Representative gross images of uteri from each group. (**C**) Relative uterine weight (uterine weight/body weight, mg/g). (**D**,**E**) Representative hematoxylin and eosin (H&E)-stained vaginal tissue sections and corresponding quantification of epithelial thickness. Arrows indicate the vaginal epithelial thickness measured. Scale bar = 60 μm. Data are presented as means ± SDs (*n* = 8 animals per group). Statistical analysis was performed using the Kruskal–Wallis nonparametric test followed by Dunn’s multiple comparison test. # *p* < 0.05, ## *p* < 0.01, and ### *p* < 0.001 vs. normal control (NC) group; * *p* < 0.05, ** *p* < 0.01, and *** *p* < 0.001 vs. menopausal control (MC) group.

As shown in Figure 8A,B, the menopausal control (MC) group exhibited significantly decreased plasma E2 concentrations and uterine weights compared to the normal control (NC) group, confirming successful induction of menopause through ovarian follicle depletion. The positive control (PC) group treated with E2 (50 μg/kg) demonstrated a marked increase in both parameters, as expected from exogenous E2 administration. WS treatment resulted in dose-dependent increases in measured circulating E2 and uterine weight, with the high-dose group (WS-H) approaching levels observed in the NC and PC groups.

To further assess estrogenic effects on estrogen-sensitive tissues, vaginal sections were examined histologically after hematoxylin and eosin (H&E) staining. The MC group showed marked thinning of the vaginal epithelium, indicative of estrogen deficiency. In contrast, both the PC and WS-treated groups showed a thicker vaginal epithelium than the MC group. Quantitative histological analysis confirmed a dose-dependent increase in epithelial thickness in WS-treated animals (Figure 8C), indicating improved tissue integrity.

Collectively, these findings suggest that WS exhibited estrogen-like activity in vivo, as reflected by increased uterine weight and improved vaginal epithelial morphology. The observed increase in circulating E2 levels was associated with WS treatment; however, the present findings should be interpreted cautiously with respect to endogenous estradiol production.

## 4. Discussion

Formulation of *Silybum marianum* as a water-soluble complex markedly enhanced its estrogenic activity. UPLC–QTOF–MS analysis confirmed the presence of the major flavonolignans, including silybin and isosilybin, after microwave-assisted processing. HPLC analysis showed higher concentrations of quantified SME-derived flavonolignans and taxifolin in the 0.20-µm-filterable aqueous fraction of WS than of SME under the tested conditions. The Total Quantified SME-derived flavonolignans and taxifolin was 231.48 ± 2.00 µg/mL for WS, compared with 15.37 ± 0.11 µg/mL for SME. While SME has been previously reported to act as a weak ERβ agonist [20,21], WS showed significant binding to both ERα and ERβ. Downstream functional responses, including phosphorylation of ERα, ERK, and AKT and ER-dependent proliferation, were characterized in MCF-7 cells, a model in which ERα predominates, and were abolished by co-treatment with the ER antagonist fulvestrant. These findings suggest that the water-soluble formulation broadens the functional engagement of *Silybum marianum* with estrogen signaling pathways. Importantly, WS-induced MCF-7 cell proliferation was comparable to that elicited by representative phytoestrogens, including genistein and daidzein, further supporting its relevance as a potential phytoestrogenic agent for managing estrogen-deficiency-related conditions [32]. Collectively, these findings support the conclusion that WS has pharmacological activity in a range comparable to that of established phytoestrogens.

**Figure A1:**
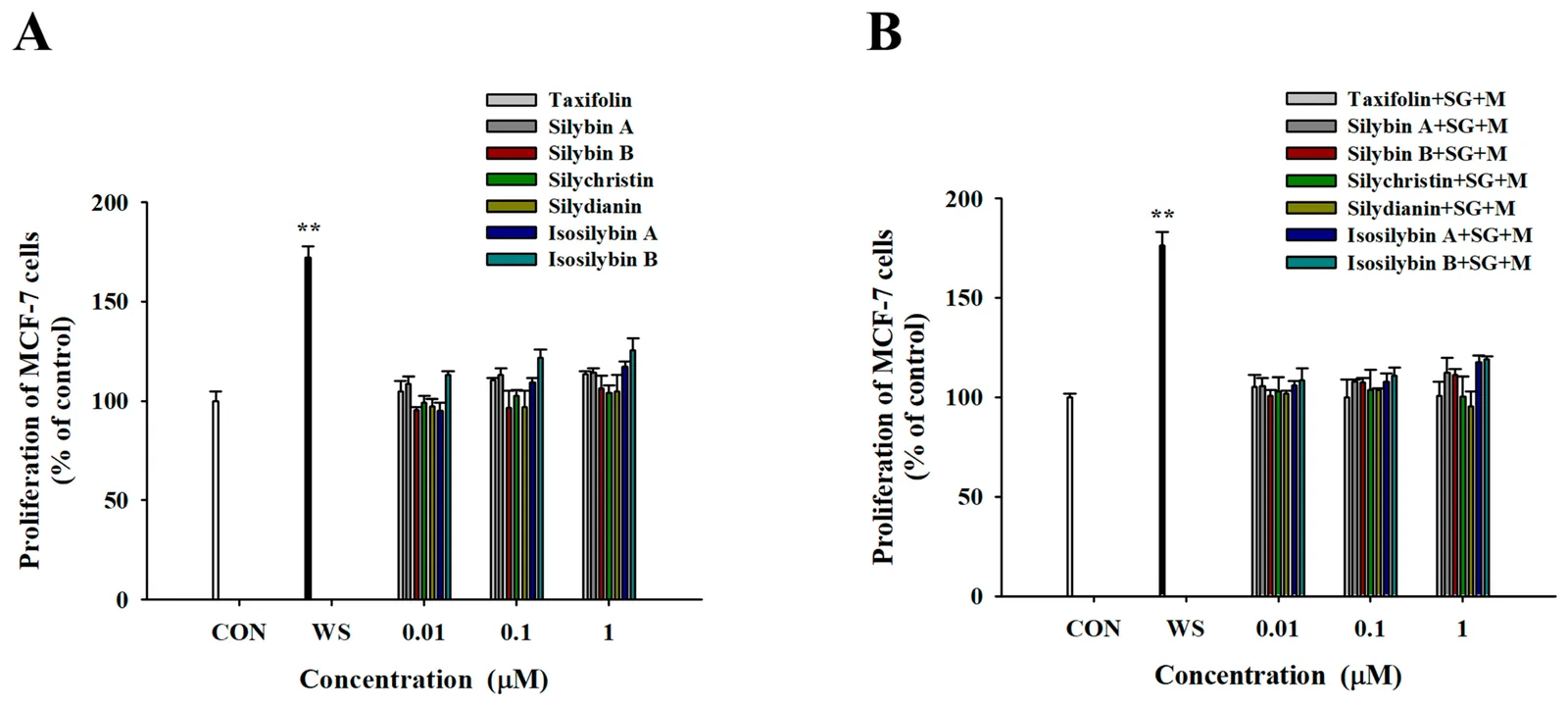
Proliferative response of MCF-7 cells to individual SME-derived flavonolignans and taxifolin (**A**) MCF-7 cells were treated with silybin A, silybin B, silychristin, silydianin, isosilybin A, isosilybin B, and taxifolin at the indicated concentrations. (**B**) Each reference compound was separately combined with steviol glycoside (SG) and subsequently subjected to microwave-assisted processing (+SG + M). Concentrations are expressed as nominal compound-equivalent concentrations calculated from the input mass of each compound. Proliferative activity was assessed using the E-screen assay. Water-soluble *Silybum marianum* complex (WS; 50 μg/mL, expressed as the nominal *Silybum marianum* extract [SME]-equivalent concentration) was included as a reference formulation. Data are expressed as means ± SDs (*n* = 4 independent experiments). Statistical significance was determined using the Kruskal–Wallis nonparametric test followed by Dunn’s multiple comparison test. ** *p* < 0.01 vs. untreated control cells (CON).

The inclusion of SG in the WS formulation further emphasizes the importance of the formulation strategy. Although SG itself exhibited no measurable estrogenic activity, microwave-assisted processing in the presence of SG markedly improved the aqueous dispersibility of the extract, as indicated by the significantly lower turbidity of WS than of both SME and microwave-treated SME (SME + M). This processing was additionally associated with changes in the compositional profile specific to the SG component, as UPLC-QTOF-MS/MS detected features putatively assigned to SG-derived metabolites in WS, while the major SME-derived flavonolignans remained detectable in WS. Importantly, neither SME alone nor its simple physical mixture with SG, regardless of microwave treatment, reproduced the estrogenic potency of WS. Consistent with this, none of the individual flavonolignans (silybin A and B, isosilybin A and B, silychristin, silydianin, and taxifolin) exhibited appreciable estrogenic activity when tested separately (Appendix A.2, Figure A1), indicating that the activity of WS cannot be attributed to any single constituent. Rather, the estrogenic efficacy of WS required the formulation process of microwave-assisted processing of SME in the presence of SG, in which the physicochemical state and compositional profile of the phytochemical matrix are modified in a manner not reproduced by simple physical mixing. Our results aligned with earlier reports showing that formulation and processing strategies can markedly enhance the biological activity of poorly water-soluble phytochemicals compared with the unprocessed materials [22,23,33].

**Figure A2:**
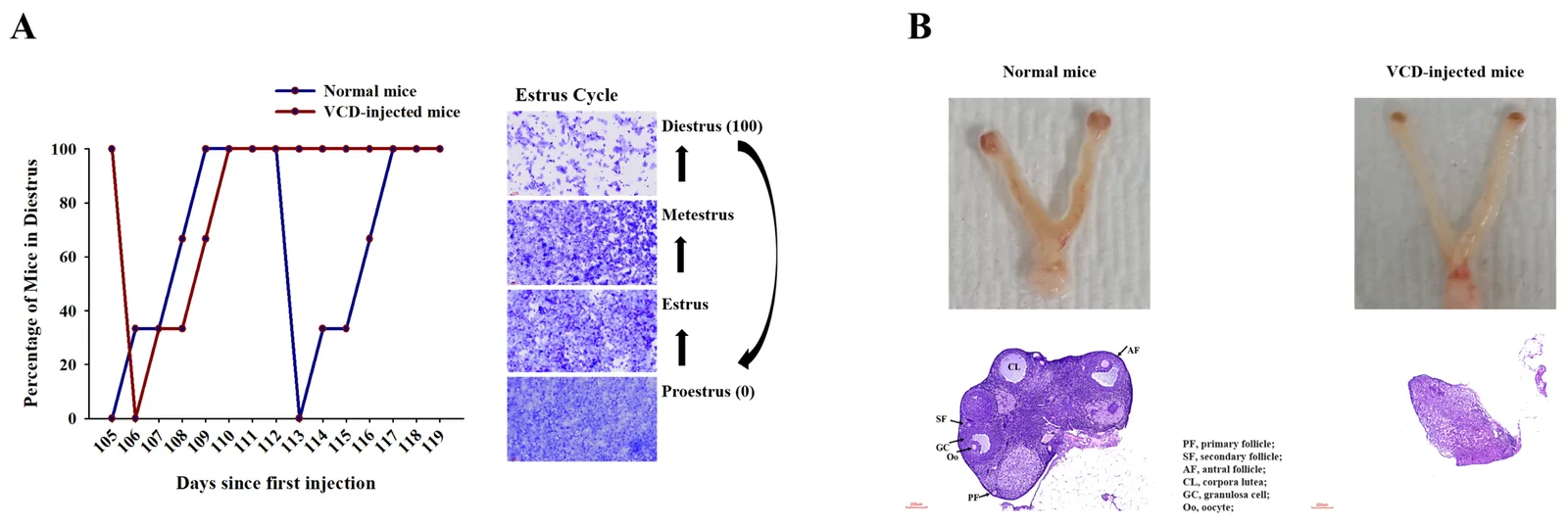
Confirmation of VCD-induced menopause in mice. (**A**) Estrous cycle monitoring showing the percentage of mice in persistent diestrus following 4-vinylcyclohexene diepoxide (VCD) administration, compared to normal cycling controls. Representative vaginal smear cytology images are provided for each stage of the estrous cycle. (**B**) Gross morphology of uteri from normal and VCD-treated mice, and representative ovarian histology (H&E staining), demonstrating depletion of primary and secondary follicles in VCD-treated ovaries, indicative of ovarian failure. Scale bar = 200 μm.

The biological relevance of these in vitro findings was validated in vivo using a VCD-induced menopausal mouse model. Successful induction of ovarian failure prior to WS treatment was confirmed by persistent diestrus, non-detectable plasma E2, elevated FSH, and depletion of ovarian follicles on histology (Appendix B.1, Figure A2). Oral administration of WS led to dose-dependent increases in measured plasma E2 levels, increased uterine weight, and vaginal epithelial thickness. The latter two endpoints are well-established indicators of estrogen-responsive tissue effects [34]. These effects were comparable to those achieved by E2 treatment, supporting the systemic estrogenic activity of WS as a phytoestrogenic formulation under estrogen-deficient conditions. As VCD-induced follicle depletion is generally considered irreversible [35], and ovarian histology was not re-examined after WS treatment, the increase in measured circulating E2 following WS administration is more plausibly attributable to WS administration itself than to recovery of ovarian follicular function. Taken together, these results indicate that WS possesses estrogenic activity both in vitro and in vivo.

Some limitations warrant consideration. First, while MCF-7 cells are widely used to investigate ERα-driven signaling due to their receptor profile, further studies using ERβ-dominant or ERβ-knockout models are necessary to clarify subtype-specific actions of WS. Second, although the VCD model reliably reproduces ovarian failure, it only partially represents the complexity of human menopause. The endpoints assessed in this study were limited to estrogen-responsive reproductive tissues, and no data were obtained on bone density, cardiovascular or metabolic outcomes, cognitive function, mammary tissue, or long-term toxicity; the present findings therefore cannot be extended to the broader spectrum of menopausal symptoms or to the long-term consequences of estrogen deficiency. Relatedly, the estrogenic activity demonstrated in MCF-7 cells and in the VCD model is not by itself evidence of a favorable safety profile, as the MCF-7 proliferation assay is designed to detect estrogenic potency rather than to assess safety, and the same ER-dependent proliferative response could in principle also stimulate other estrogen-sensitive tissues such as the breast and endometrium. The long-term effects of WS on these tissues therefore remain a critical open question rather than an implicit safety endorsement. Third, HPLC analysis indicated differences in the aqueous concentrations of major flavonolignans between SME and WS. However, formal equilibrium solubility, for example, as determined by the shake-flask method, as well as formulation stability and bioavailability, were not directly evaluated in this study. In addition, plasma estradiol was quantified by a commercial ELISA without LC-MS/MS confirmation; because the manufacturer’s cross-reactivity validation for the kit used in this study did not include the non-steroidal phytochemical constituents of WS, the possibility of assay interference cannot be fully excluded. Finally, although we demonstrated ER binding, proliferative effects, and rapid intracellular signaling, nuclear transactivation, receptor subtype specificity, and potential involvement of GPER remain to be investigated. Future studies should therefore address these mechanistic aspects, together with pharmacokinetics, long-term efficacy, and safety.

## 5. Conclusions

In summary, formulation of *Silybum marianum* as a water-soluble complex using microwave-assisted processing with steviol glycoside (SG) markedly enhanced its estrogenic activity. This effect was not reproduced by the individual flavonolignans or by a simple physical combination of *Silybum marianum* extract (SME) with SG, highlighting that the enhanced estrogenic activity of WS requires the combined formulation process—microwave-assisted processing of SME in the presence of SG rather than arising from SME, SG, microwave-assisted processing alone, or the individual flavonolignans. WS demonstrated direct interactions with both ERα and ERβ, activated rapid ER signaling in ERα-positive MCF-7 cells, and elicited estrogen-like effects in a VCD-induced menopausal mouse model, including increased measured circulating estradiol levels, increased uterine weight, and reversal of vaginal epithelial atrophy. Collectively, these findings support WS as a promising phytoestrogenic candidate for further evaluation in the context of estrogen deficiency. Before any comparison with MHT can be made, further studies evaluating its pharmacokinetics, long-term effects on estrogen-sensitive tissues such as breast and endometrium, and overall safety profile are warranted. 

## Figures and Tables

**Table 1:** MRM conditions for Rebaudioside A and stevioside.

Compound	Precursor (*m*/*z*)	Product (*m*/*z*)	DP (V)	CE (V)	CXP (V)
Rebaudioside A	965.354	803.4	−140	−54	−35
	965.354	641.4	−140	−88	−25
Stevioside	803.301	641.2	−125	−34	−33
	803.301	479.3	−125	−84	−19

**Table 2:** Putative identification of chemical constituents detected in water-soluble *Silybum marianum* complex (WS) and *Silybum marianum* extract (SME) by UPLC-ESI-QTOF-MS in positive ion mode.

	R_t_ (min)		Molecular Formula	Ion Form	*m*/*z* (Da)	MS/MS Fragments (*m*/*z*)	Library Score		Putative Compound
No.	WS	SME					WS	SME	
1	0.92	0.91	C_8_H_20_NO_6_P	[M + H]+	258.1109	184.0735, 125.0011, 104.1073, 86.0970	98.8	96.7	Glycerophosphocholine
2	8.47	8.47	C_27_H_30_O_16_	[M + H]+	611.1653	303.0499	93.8	96.5	Quercetin 3-rutinoside (Rutin)
3	8.57	8.57	C_15_H_12_O_7_	[M + H]+	305.0646	287.0556, 259.0599, 231.0645, 153.0180	98.3	98.4	Dihydroquercetin (Taxifolin)
4	8.72	8.72	C_21_H_20_O_12_	[M + H]+	465.1058	303.0523	96.3	100	Hyperoside(Hyperin)
5	10.12	10.12	C_21_H_22_O_10_	[M + H]+	435.1291	273.0762, 153.0191, 91.0531	98.2	98.2	Naringenin-7-O-glucoside
6	10.16	10.15	C_15_H_12_O_6_	[M + H]+	289.070	243.0664, 215.0707, 153.0189, 149.0239, 107.0491	95.6	93.6	Dihydrokaempferol
7	11.42	11.38	C_25_H_22_O_10_	[M + H]+	483.1281	453.1067, 407.1129, 195.0294, 153.0183	96.3	96.4	Silychristin
8	12.18	12.19	C_15_H_12_O_6_	[M + H]+	289.0723	163.0397, 153.0181	92.8	97	Eriodictyol
9	12.18	-	C_44_H_70_O_23_	[M + NH_4_]+	984.4665	649.2185, 325.1140, 163.0582	90.7		RebaudiosideA + NH_3_
10	12.47	12.46	C_15_H_10_O_7_	[M + H]+	303.0495	229.0500, 165.0194, 153.0187, 137.0242	99.8	96.5	Quercetin
11	13.96	13.95	C_15_H_12_O_5_	[M + H]+	273.0772	153.0191, 147.0446,119.0504, 91.0543	100	100	Naringenin
12	14.01	13.99	C_25_H_22_O_10_	[M + H]+	483.1275	437.1221, 195.0289, 153.0177, 131.0495	89.7	92.4	Silybin
13	14.48	-	C_44_H_70_O_23_	[M + H]+	967.4396	325.1129, 163.0610	98.1		RebaudiosideA
14	14.68	14.66	C_25_H_22_O_10_	[M + H]+	483.1274	419.1126, 311.0555, 257.0452, 162.0678, 153.0176, 147.0444, 131.0498	96.2	94.2	IsosilybinA
15	15.47	-	C_12_H_22_O_11_	[M + H]+	325.1143	85.0294, 69.0348	91.7		6.alpha.-Mannobiose
16	15.83	-	C_20_H_30_O_3_	[M + H]+	319.2266	301.2157, 273,2212, 255.2110	95.9		Steviol
17	15.83	-	C_32_H_50_O_13_	[M + Na]+	665.3160	665.3136	89.5		Steviolbioside
18	16.74	-	C_20_H_30_O_3_	[M + H]+	319.2266	301.2157, 273,2212, 255.2110	96.5		Isosteviol
19	27.19	27.19	C_23_H_48_NO_7_P	[M + H]^+^	482.3260	464.3248, 184.0747, 104.1080	96.1	96	Lyso PC (15:0)
20	27.66	27.65	C_26_H_52_NO_7_P	[M + H]^+^	522.3475	184.0735, 104.1075	91.7	86.1	Lyso PC (18:1)
21	28.61	28.61	C_24_H_50_NO_7_P	[M + H]^+^	496.3391	478.3298, 184.0722, 104.1066	91.5	92.1	Lyso PC (16:0)
22	29.12	29.1	C_26_H_54_NO_7_P	[M + H]^+^	524.3708	506.3540, 184.0745, 104.1067, 86.0958	97.4	90.2	Lyso PC (18:0)
23	-	33.98	C_44_H_80_NO_8_P	[M + H]^+^	782.5688	184.0720		87	PC(18:2(9Z,12Z)/18:2(9Z,12Z))
24	-	34.39	C_41_H_74_NO_8_P	[M + H]^+^	740.5239	599.5045		94.5	PE(18:2(9Z,12Z)/18:2(9Z,12Z))

**Table 3:** Quantitative characterization of SME- and SG-derived constituents in WS.

Source	Marker Compound	WS Batch 1 (*n* = 3)	WS Batch 2 (*n* = 3)	WS Batch 3 (*n* = 3)	Overall Mean ± SD Across Batches
SME-derived flavonolignans and taxifolin	Silybin A +Isosilybin B (µg/mL)	18.30 ± 0.18 **^†^**	18.49 ± 0.29 **^†^**	18.02 ± 0.27 **^†^**	18.27 ± 0.24
	Silybin B (µg/mL)	21.91 ± 0.25	22.14 ± 0.37	21.63 ± 0.26	21.89 ± 0.26
	Silychristin (µg/mL)	101.88 ± 0.47	102.27 ± 0.57	99.98 ± 0.79	101.38 ± 1.23
	Silydianin (µg/mL)	24.84 ± 0.33	24.97 ± 0.09	23.39 ± 0.82	24.40 ± 0.87
	Isosilybin A (µg/mL)	47.94 ± 0.41	48.48 ± 0.77	47.38 ± 0.74	47.93 ± 0.55
	Taxifolin (µg/mL)	15.37 ± 0.11	15.35 ± 0.09	14.94 ± 0.09	15.22 ± 0.24
Total quantified SME-derived flavonolignans and taxifolin (µg/mL)		230.68 ± 1.45	231.48 ± 2.00	225.32 ± 1.59	229.16 ± 3.35
SG	Stevioside (mg/mL)	0.689 ± 0.015	0.653 ± 0.011	0.676 ± 0.016	0.673 ± 0.018
	Rebaudioside A (mg/mL)	0.611 ± 0.008	0.651 ± 0.017	0.582 ± 0.009	0.598 ± 0.015
Total Quantified stevioside and rebaudioside A (mg/mL)		1.258 ± 0.007	1.300 ± 0.022	1.258 ± 0.007	1.271 ± 0.025

Values are presented as means ± SDs of three independently prepared WS batches (*n* = 3); each batch was analyzed by triplicate HPLC or LC–MS/MS injections, in which multiple analytes were quantified simultaneously. WS was dispersed in DW at a nominal SME-equivalent loading of 1 mg/mL (nominal SG concentration, 2 mg/mL) and filtered through a 0.20-µm hydrophilic PTFE syringe filter before analysis. Values are expressed as µg/mL (SME-derived analytes) or mg/mL (SG-derived analytes) in the 0.20-µm filtrate. **^†^** Silybin A and isosilybin B co-eluted under the chromatographic conditions used and were quantified as a combined peak.

**Table 4:** HPLC quantification of SME-derived flavonolignans and taxifolin in filtered aqueous fractions of SME and WS.

SME-Derived Flavonolignans and Taxifolin	SME (µg/mL in Original Filtrate)	WS (µg/mL in Original Filtrate)
Silybin A +Isosilybin B	ND **^†, ††^**	18.49 ± 0.29 **^†^**
Silybin B	ND **^††^**	22.14 ± 0.37
Silychristin	7.06 ± 0.06	102.27 ± 0.57
Silydianin	2.76 ± 0.08	24.97 ± 0.09
Isosilybin A	ND **^††^**	48.48 ± 0.77
Taxifolin	5.50 ± 0.04	15.35 ± 0.09
Total Quantified SME-derived flavonolignans and taxifolin	15.37 ± 0.11	231.48 ± 2.00

Values are presented as means ± SDs from three independently prepared dispersions (*n* = 3), each analyzed by a single HPLC injection. *Silybum marianum* extract (SME) and water-soluble *Silybum marianum* complex (WS) were dispersed in DW at a nominal SME-equivalent loading of 1 mg/mL and filtered through a 0.20-µm hydrophilic PTFE syringe filter before HPLC analysis. Values are expressed as µg/mL in the original filtrate. **^†^** Silybin A and isosilybin B co-eluted under the chromatographic conditions used and were quantified as a combined peak. **^††^** Total represents the sum of quantified markers. ND, not detected.

**Figure A3:**
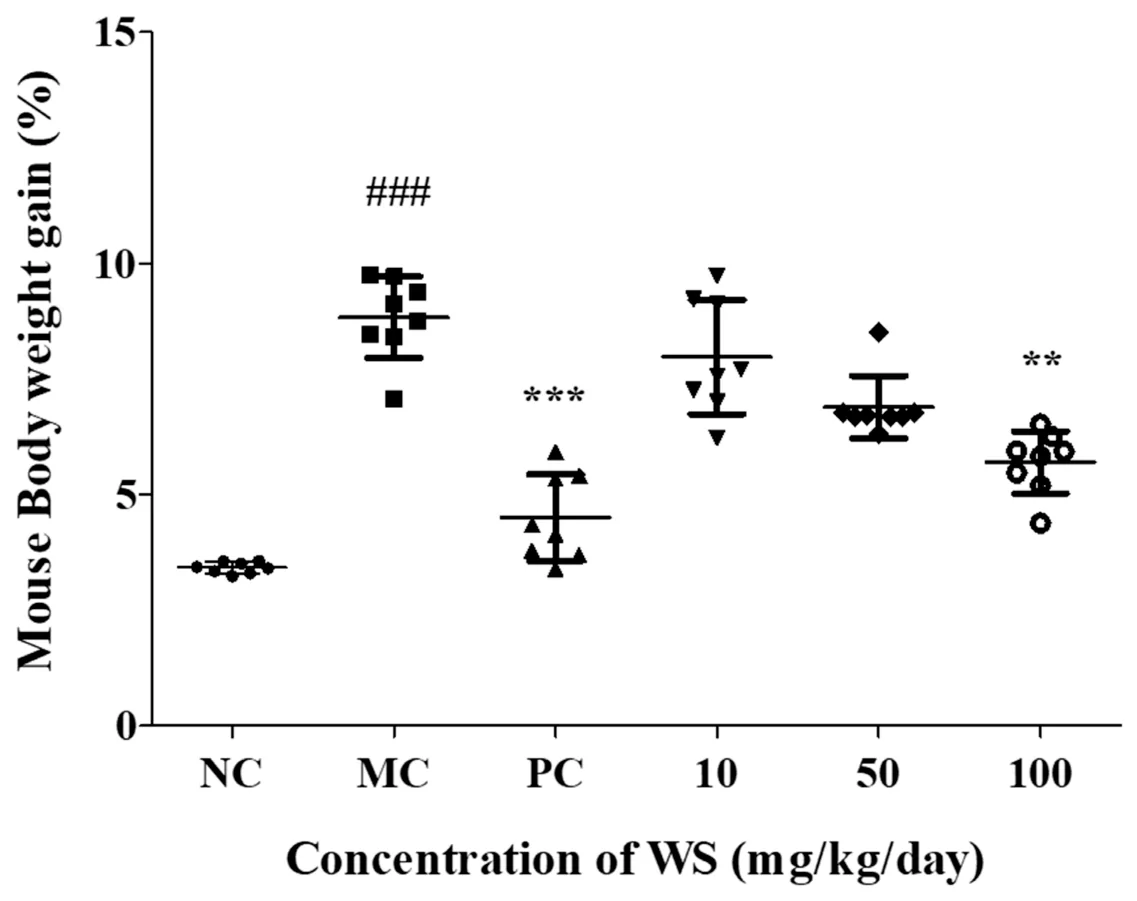
Body weight gain in mice during WS treatment. Body weight gain (%) was calculated from initial and final body weight measurements (Table A4) in the normal control (NC) group, menopausal control (MC) group, positive control (PC; estradiol-treated) group, and water-soluble *Silybum marianum* complex (WS)-treated groups (10, 50, and 100 mg/kg/day). Data are presented as individual values with group means ± SDs (*n* = 8 animals per group). Statistical analysis was performed using the Kruskal–Wallis nonparametric test followed by Dunn’s multiple comparison test. ** *p* < 0.01, *** *p* < 0.001 vs. MC; ### *p* < 0.001 vs. NC.

## Data Availability

The original data presented in this study are included in the article and the Supplementary Materials. Further inquiries can be directed to the corresponding author. The original microscopic images, original Western blot images, and statistical analysis results are provided in the Supplementary Materials.

## Supplementary Materials

The following supporting information can be downloaded at: https://www.mdpi.com/article/10.3390/nu18203388/s1, File S1: Original microscopic images; File S2: Original Western blot images; File S3: Statistical analysis results.

## Abbreviations

The following abbreviations are used in this manuscript:

BrdU	5-Bromo-2′-deoxyuridine
CD-FBS	Charcoal-Dextran–Stripped Fetal Bovine Serum
E2	Estradiol
ELISA	Enzyme-Linked Immunosorbent Assay
ER	Estrogen Receptor
FBS	Fetal Bovine Serum
FP	Fluorescence Polarization
H&E	Hematoxylin and Eosin
NC	Normal Control
MC	Menopausal Control
MHT	Menopausal hormone therapy
PC	Positive Control
SME	*Silybum marianum* extract
SG	Steviol glycoside
SME + SG	Physical mixture of SME and SG
SME + M	Microwave-treated SME
VCD	4-Vinylcyclohexene Diepoxide
WS	Water-soluble *Silybum marianum* complex

## Appendix A. In Vitro Supplementary Data

### Appendix A.1. Additional HPLC Characterization Data for Filterable Aqueous Fractions of SME and WS

**Table A1:** Concentration- and time-dependent HPLC quantification of individual SME-derived flavonolignans and taxifolin in the 0.20-µm-filterable aqueous fractions of SME and WS.

Sampling Day	Nominal SME-Equivalent Concentration (mg/mL)	Sample	Silybin A + Isosilybin B (µg/mL)	Silybin B (µg/mL)	Silychristin (µg/mL)	Silydianin (µg/mL)	Isosilybin A (µg/mL)	Taxifolin (µg/mL)	Total Quantified Flavonolignans and Taxifolin (µg/mL)
Day 1	1	WS	17.63 ± 0.13	22.41 ±0.14	92.27 ± 0.34	20.44 ± 0.10	39.75 ± 0.34	14.97 ± 0.46	207.5 ± 0.7
		SME	ND	ND	6.63 ± 0.04	0.91 ± 0.02	ND	5.24 ± 0.05	12.8 ± 0.1
	5	WS	93.85 ± 3.79	109.52 ± 1.07	499.30 ± 3.15	104.86 ± 1.09	204.19 ± 2.13	77.66 ± 0.33	1089.4 ± 11.0
		SME	0.02 ± 0.01	3.45 ± 0.04	32.62 ± 0.15	5.15 ± 1.17	3.94 ± 0.07	27.59 ± 0.14	72.8 ± 1.3
	10	WS	196.21 ± 1.39	218.74 ± 4.24	1005.36 ± 0.60	211.63 ± 0.31	404.20 ± 0.04	158.55 ± 0.09	2194.7 ± 5.6
		SME	2.62 ± 0.05	6.45 ± 0.10	62.15 ± 0.23	9.06 ± 0.03	9.03 ± 0.08	55.50 ± 0.04	144.8 ± 0.5
Day 2	1	WS	15.84 ± 0.10	21.62 ± 0.12	91.50 ± 0.19	20.15 ± 0.05	38.20 ± 0.27	14.78 ± 0.04	202.1 ± 0.7
		SME	ND	ND	6.48 ± 0.02	1.31 ± 0.01	ND	5.06 ± 0.04	12.8 ± 0.1
	5	WS	93.71 ± 2.66	110.00 ± 1.41	498.68 ± 1.66	103.65 ± 0.31	204.23 ± 3.05	78.10 ± 0.08	1088.4 ± 5.9
		SME	0.05 ± 0.01	3.53 ± 0.02	32.40 ± 0.02	6.51 ± 0.02	4.01 ± 0.04	27.55 ± 0.05	74.1 ± 0.1
	10	WS	196.11 ± 0.37	219.89 ± 0.24	1005.39 ± 1.09	208.58 ± 0.13	404.4 ± 0.55	158.69 ± 0.08	2193.1 ± 2.4
		SME	2.66 ± 0.13	6.56 ± 0.15	59.87 ± 0.42	16.09 ± 0.09	9.15 ± 0.31	55.06 ± 0.10	149.4 ± 1.2
Day 3	1	WS	17.11 ± 0.74	22.32 ± 0.19	92.29 ± 0.09	20.12 ± 0.02	39.97 ± 1.27	14.78 ± 0.04	206.6 ± 2.1
		SME	ND	ND	6.41 ± 0.02	1.29 ± 0.01	ND	4.79 ± 0.03	12.5 ± 0.1
	5	WS	95.34 ± 0.61	114.18 ± 0.55	506.86 ± 1.94	104.62 ± 1.07	213.78 ± 1.60	78.00 ± 0.61	1112.8 ± 5.2
		SME	0.01 ± 0.01	3.49 ± 0.01	32.28 ± 0.13	6.53 ± 0.04	3.95 ± 0.03	27.34 ± 0.06	73.6 ± 0.2
	10	WS	196.44 ± 0.47	220.10 ± 0.51	1006.52 ± 2.47	207.74 ± 0.87	408.30 ± 1.09	158.51 ± 0.10	2197.6 ± 5.4
		SME	2.48 ± 0.04	6.39 ± 0.04	59.15 ± 0.14	15.84 ± 0.06	8.77 ± 0.12	55.00 ± 0.05	147.6 ± 0.5

SME and WS dispersions were maintained in DW at room temperature for 1, 2, or 3 days and filtered through a 0.20-µm hydrophilic PTFE syringe filter immediately before HPLC analysis. All other sample preparation, HPLC, and data-processing procedures are as described in Section 2.3. Values are expressed as µg/mL in the original filtrate and presented as means ± SDs from three independently prepared dispersions (*n* = 3), each analyzed by a single HPLC injection. Silybin A and isosilybin B co-eluted and were quantified as a combined peak. Total represents the sum of all quantified marker compounds. ND, not detected.

**Table A2:** Relative recovery of individual marker compounds in the filterable aqueous fraction of WS relative to WS in 100% MeOH.

SME-Derived Flavonolignans and Taxifolin	WS (µg/mL in 100% MeOH)	WS (µg/mL in DW Filtrate)	Relative Recovery (%)
Silybin A +Isosilybin B	18.03 ± 0.03	17.11 ± 0.74	94.87 ± 3.99
Silybin B	22.86 ± 0.06	22.32 ± 0.19	97.64 ± 0.57
Silychristin	98.24 ± 0.14	92.29 ± 0.09	93.95 ± 0.08
Silydianin	22.59 ± 0.04	20.12 ± 0.02	89.05 ± 0.21
Isosilybin A	41.41 ± 0.06	39.97 ± 1.27	96.51 ± 2.97
Taxifolin	15.95 ± 0.03	14.78 ± 0.04	92.67 ± 0.26
Total Quantified SME-derived flavonolignans and taxifolin	219.09 ± 0.33	206.6 ± 2.1	94.30 ± 0.84

Freeze-dried WS powder was dissolved in 100% MeOH or dispersed in DW at a nominal SME-equivalent concentration of 1 mg/mL. The DW dispersion was maintained at room temperature for 3 days; both the MeOH solution and DW dispersion were filtered through a 0.20-µm hydrophilic PTFE syringe filter immediately before HPLC analysis. Relative recovery (%) was calculated as (*C_DW filtrate_/C_MeOH_*) × 100, with WS in 100% MeOH defined as the reference (100%). Values are presented as means ± SDs from three independently prepared MeOH solutions or DW dispersions of the same WS batch (*n* = 3), each analyzed by a single HPLC injection. Silybin A and isosilybin B co-eluted and were quantified as a combined peak.

## Appendix B. In Vivo Supplementary Data

### Appendix B.1. Validation of VCD-Induced Menopausal Mouse Model

**Table A3:** Serum E2 and ovarian follicle counts in normal and VCD-induced menopausal mice.

Plasma	E2 (pg/mL)	FSH (μg/mL)
Normal mice	26.67 ± 3.50	1.00 ± 0.12
VCD-injected mice	Non-detectable	1.47 ± 0.02 **

Data are expressed as means ± SDs (n = 8 animals per group). Plasma estradiol (E2) and follicle-stimulating hormone (FSH) concentrations were measured to assess the VCD-induced menopausal phenotype. E2 concentrations in VCD-treated mice were below the assay detection limit. Statistical analysis was performed using the Kruskal–Wallis test followed by Dunn’s multiple-comparison test. ** *p* < 0.01 vs. normal mice.

### Appendix B.2. Body Weight Changes in Menopausal Mice Treated with WS

**Table A4:** Body weight changes in mice during WS treatment.

Group	Initial Weight (g)	Final Weight (g)
NC	23.98 ± 0.49	24.80 ± 0.52
MC	24.14 ± 0.49	26.27 ± 0.45
PC	24.08 ± 0.35	25.16 ± 0.49
WS-10 mg/kg/day	24.89 ± 0.88	26.90 ± 1.21
WS-50 mg/kg/day	25.04 ± 0.52	26.65 ± 0.65
WS-100 mg/kg/day	24.55 ± 0.51	25.95 ± 0.44

Body weight was measured at the beginning and end of the experimental period in the normal control (NC) group, menopausal control (MC) group, positive control (PC; estradiol-treated) group, and water-soluble *Silybum marianum* complex (WS)-treated groups (10, 50, and 100 mg/kg/day). Data are expressed as means ± SDs (*n* = 8 animals per group).
