# Supplementary material for: Enhanced Estrogenic Activity in Water-Soluble Silybum marianum Complex via Microwave-Assisted Steviol Glycoside-Based Formulation
Source: Nutrients. 2026 Oct 15;18(20):3388. doi: 10.3390/nu18203388 (PMC13708382; doi:10.3390/nu18203388)

Water-soluble *silybum marianum* complex (*in vivo*)

Figure 8

Figure 7 – Vaginal tissue
(Normal Control; NC)

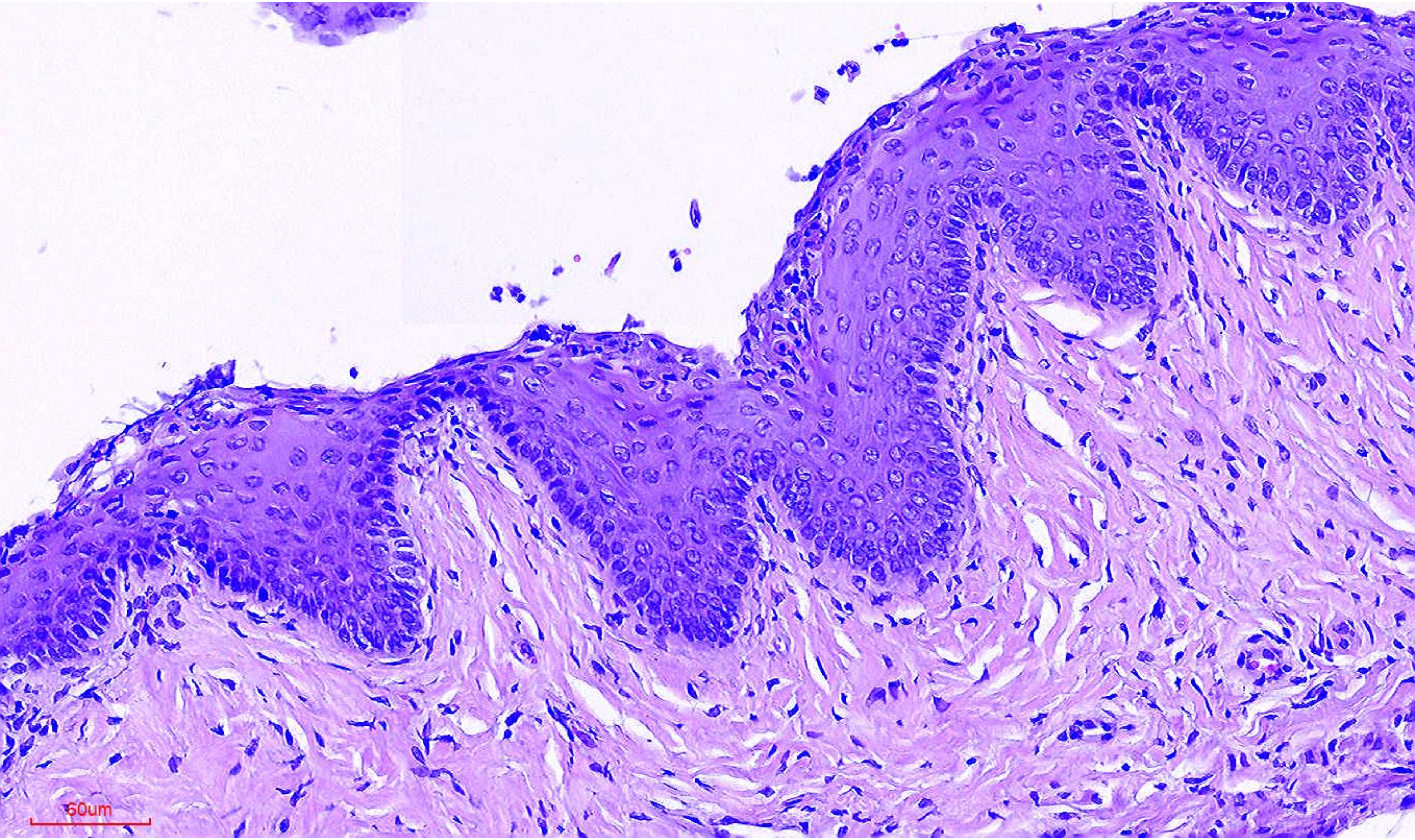

Figure 7 – Vaginal tissue (Menopause Control, MC)

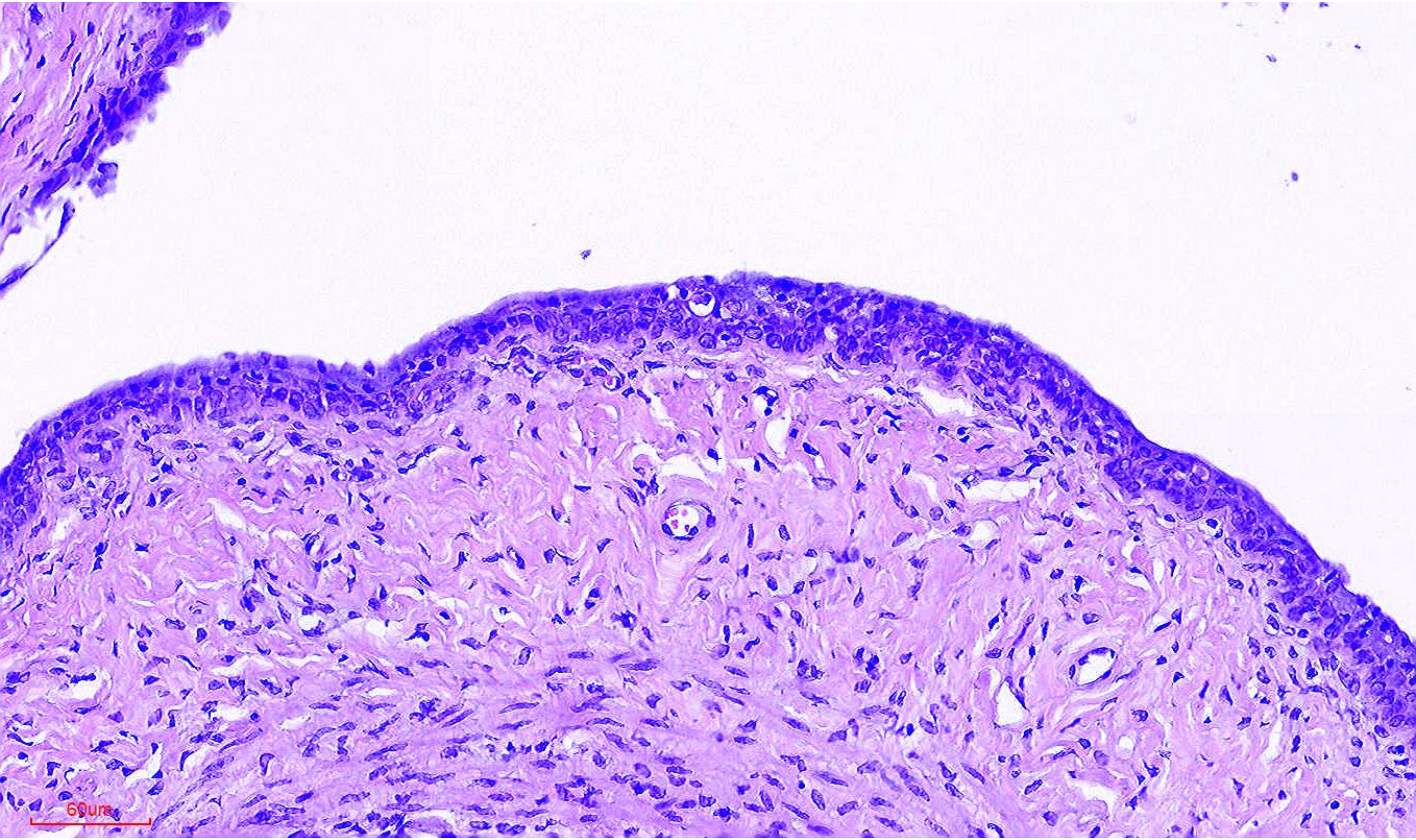

Figure 7 – Vaginal tissue
(Positive Control, PC)

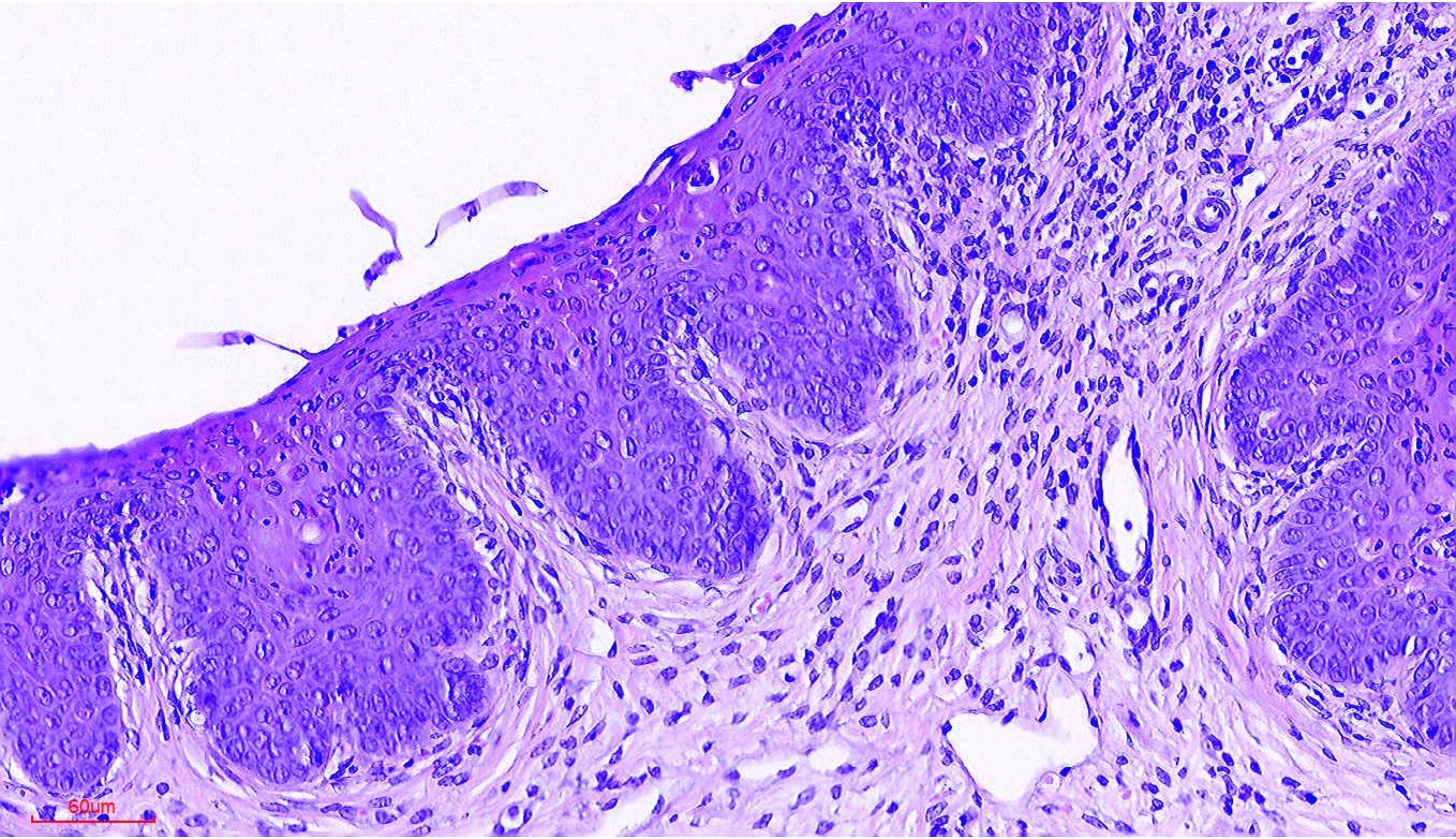

Figure 7 – Vaginal tissue
(WS; 10 mg/kg/day)

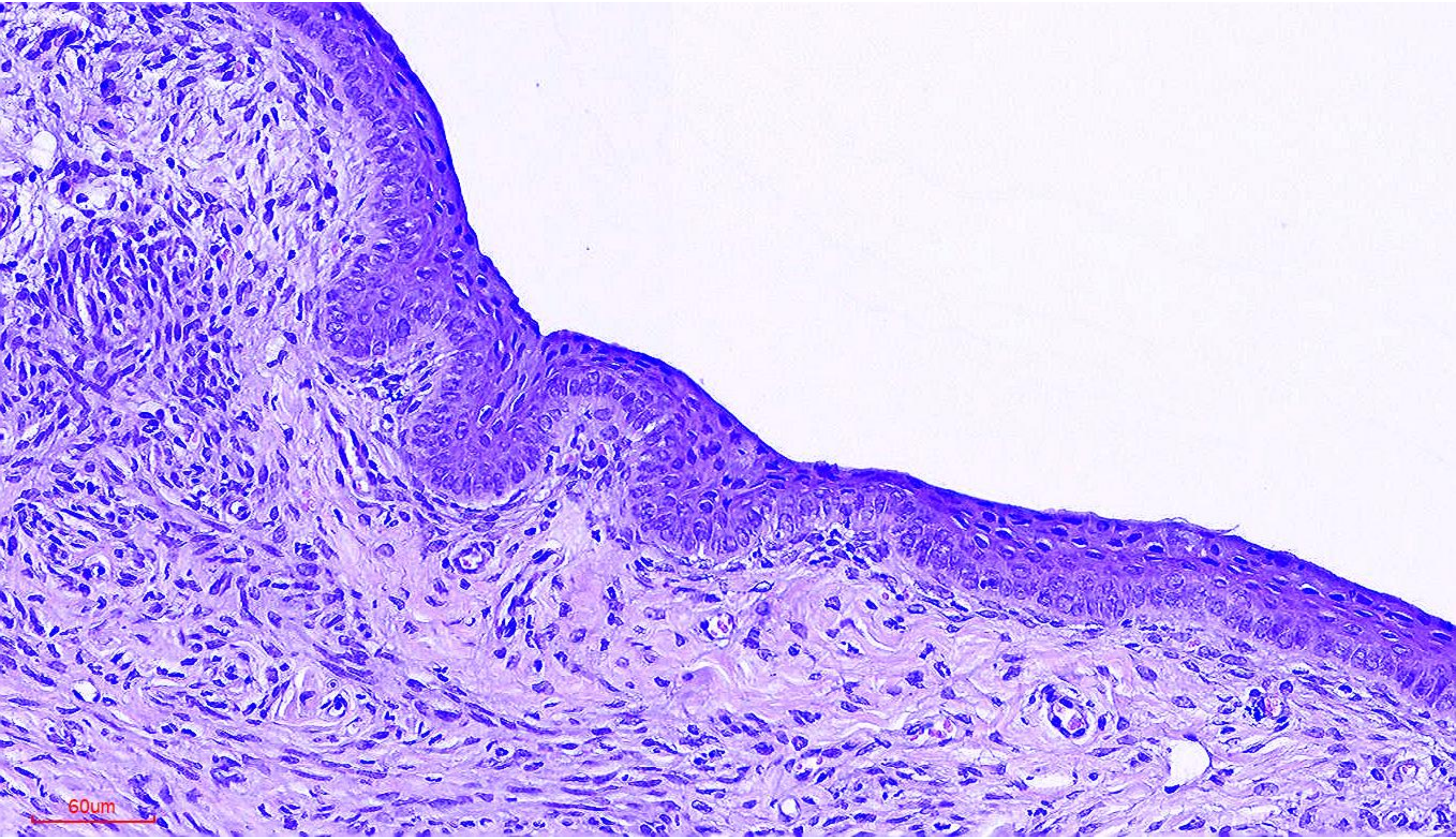

Figure 7 – Vaginal tissue
(WS; 50 mg/kg/day)

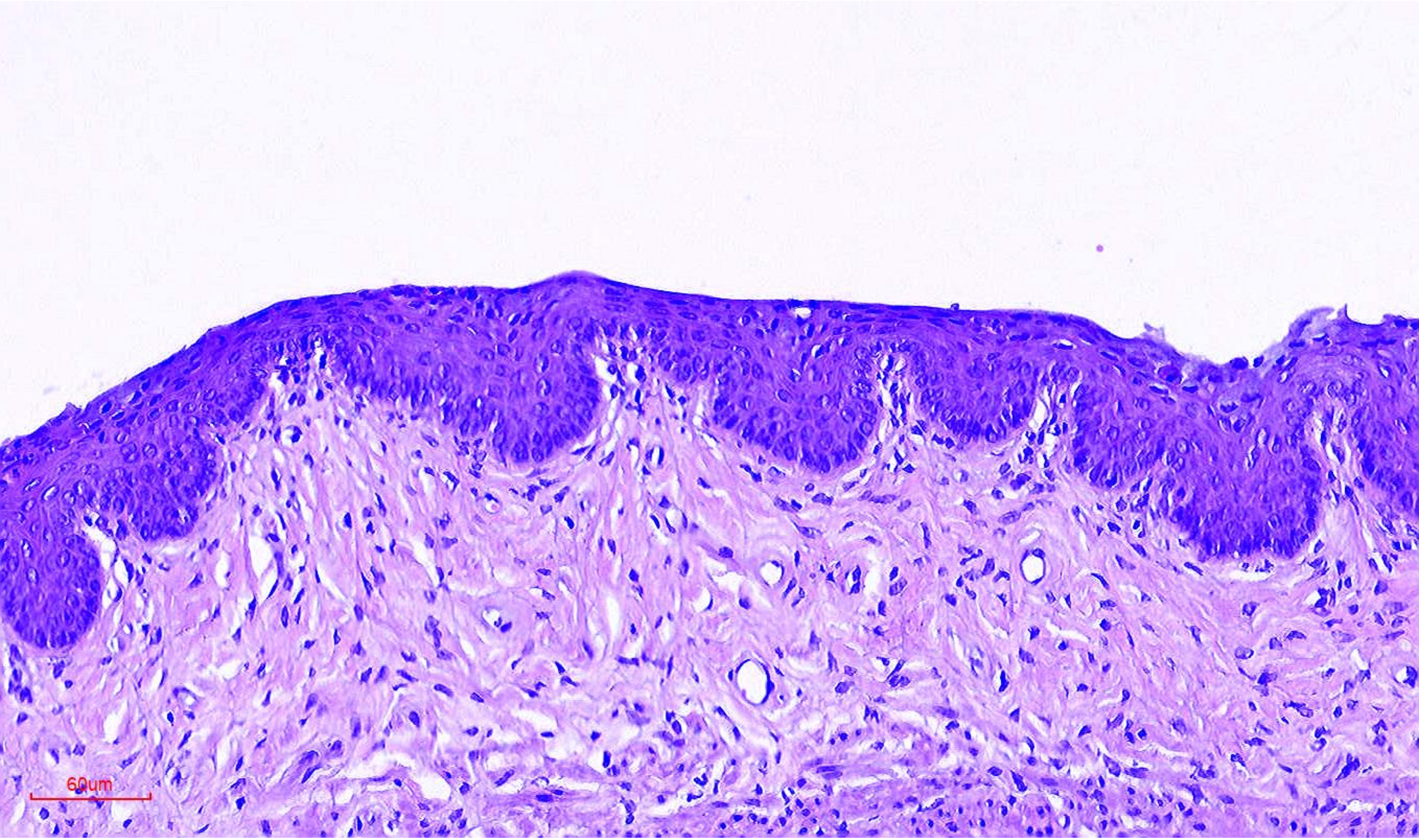

Figure 7 – Vaginal tissue
(WS; 100 mg/kg/day)

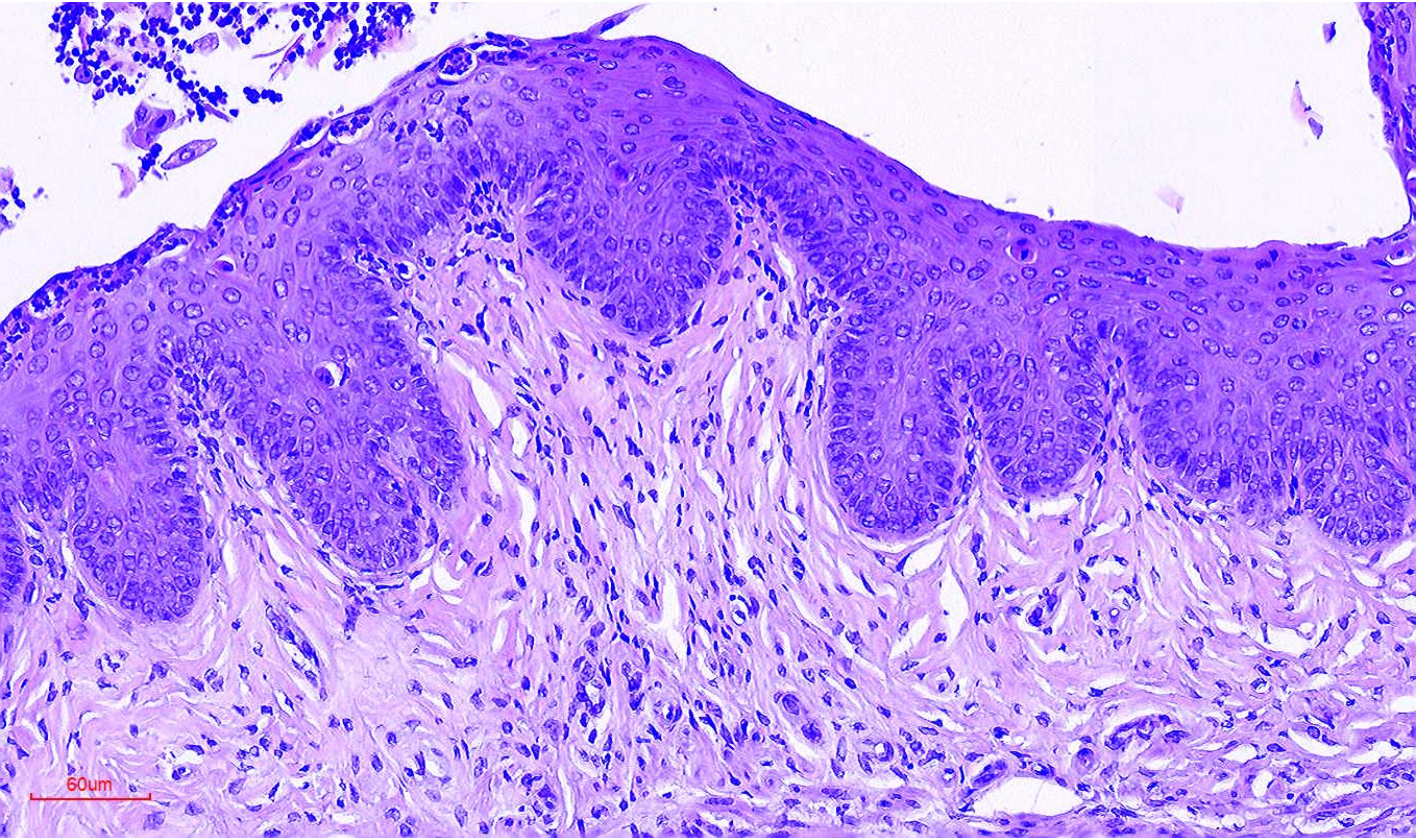

Figure B1

Figure B1 – Estrus Cycle, Vaginal smear (Proestrus)

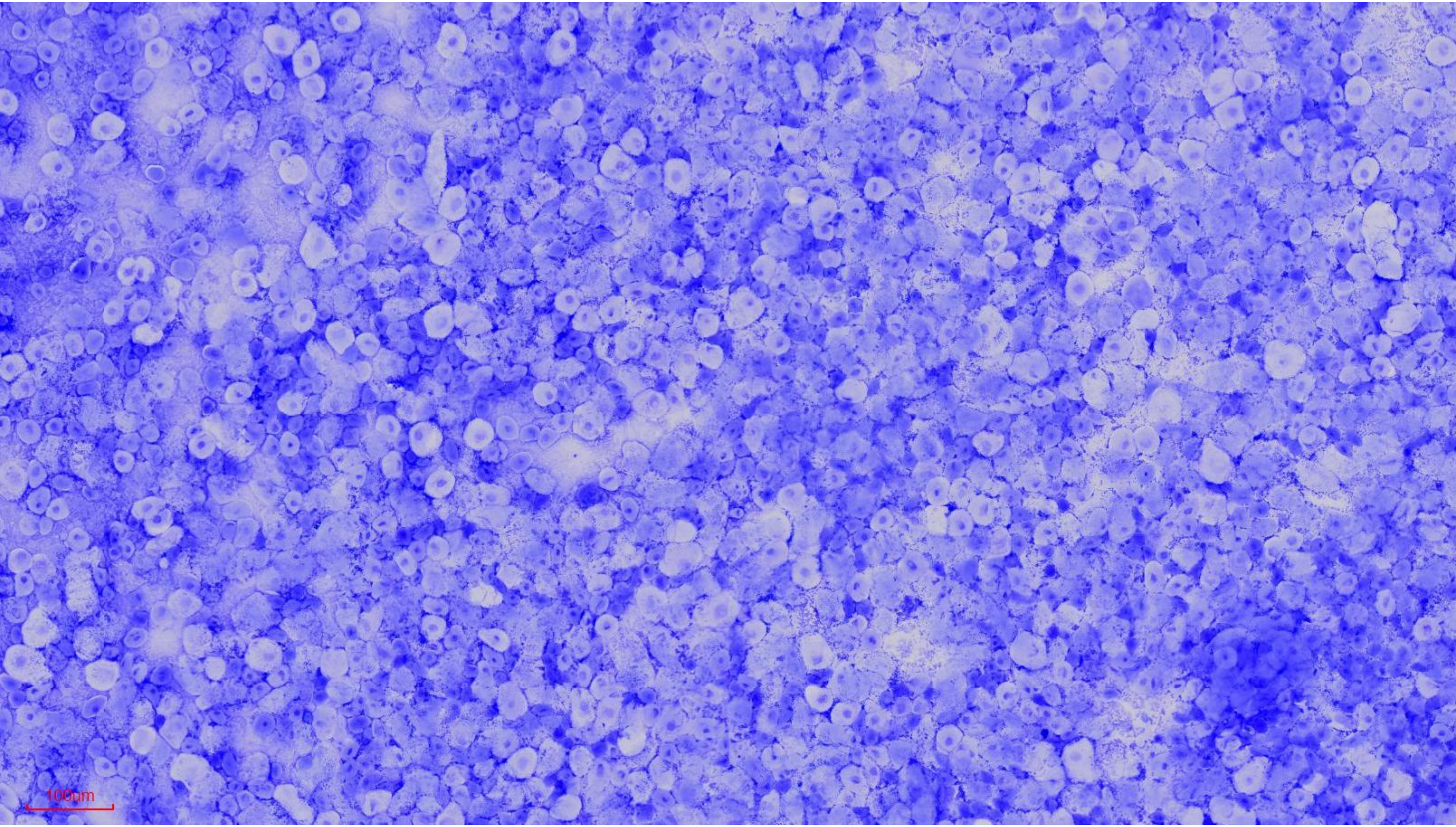

Figure B1 – Estrus Cycle, Vaginal smear (Estrus)

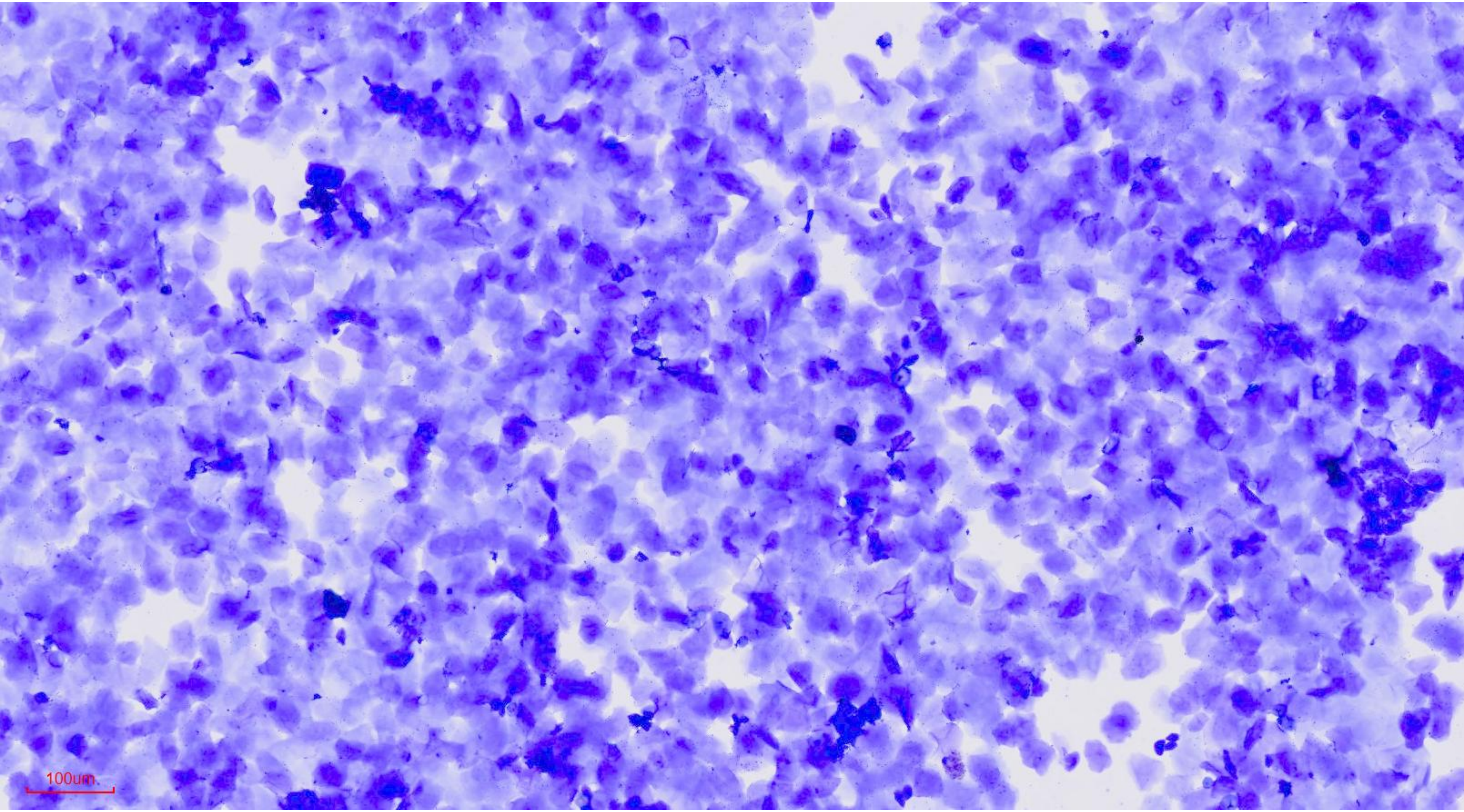

Figure B1 – Estrus Cycle, Vaginal smear
(Metestrus)

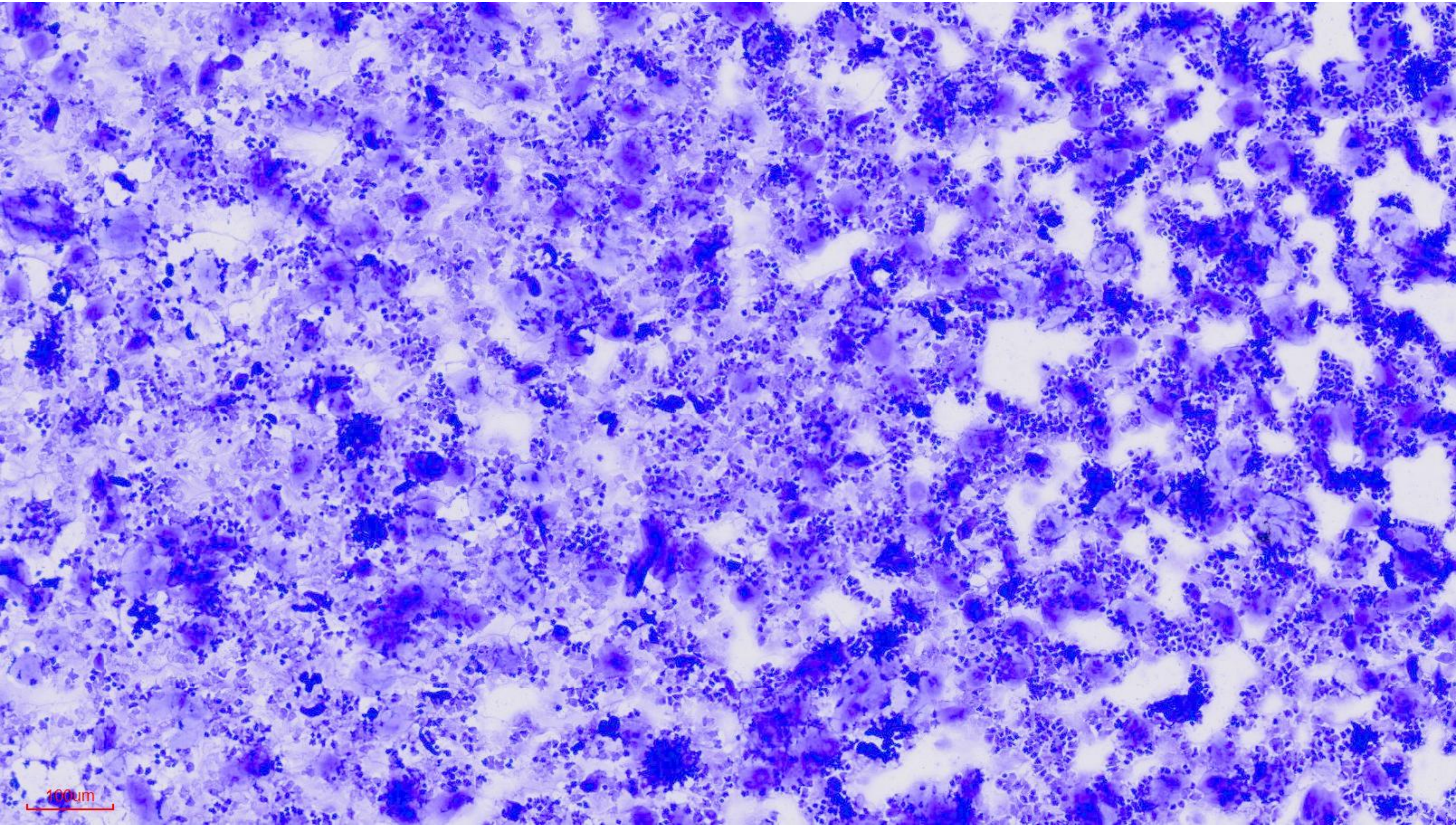

Figure B1 – Estrus Cycle, Vaginal smear (Diestrus)

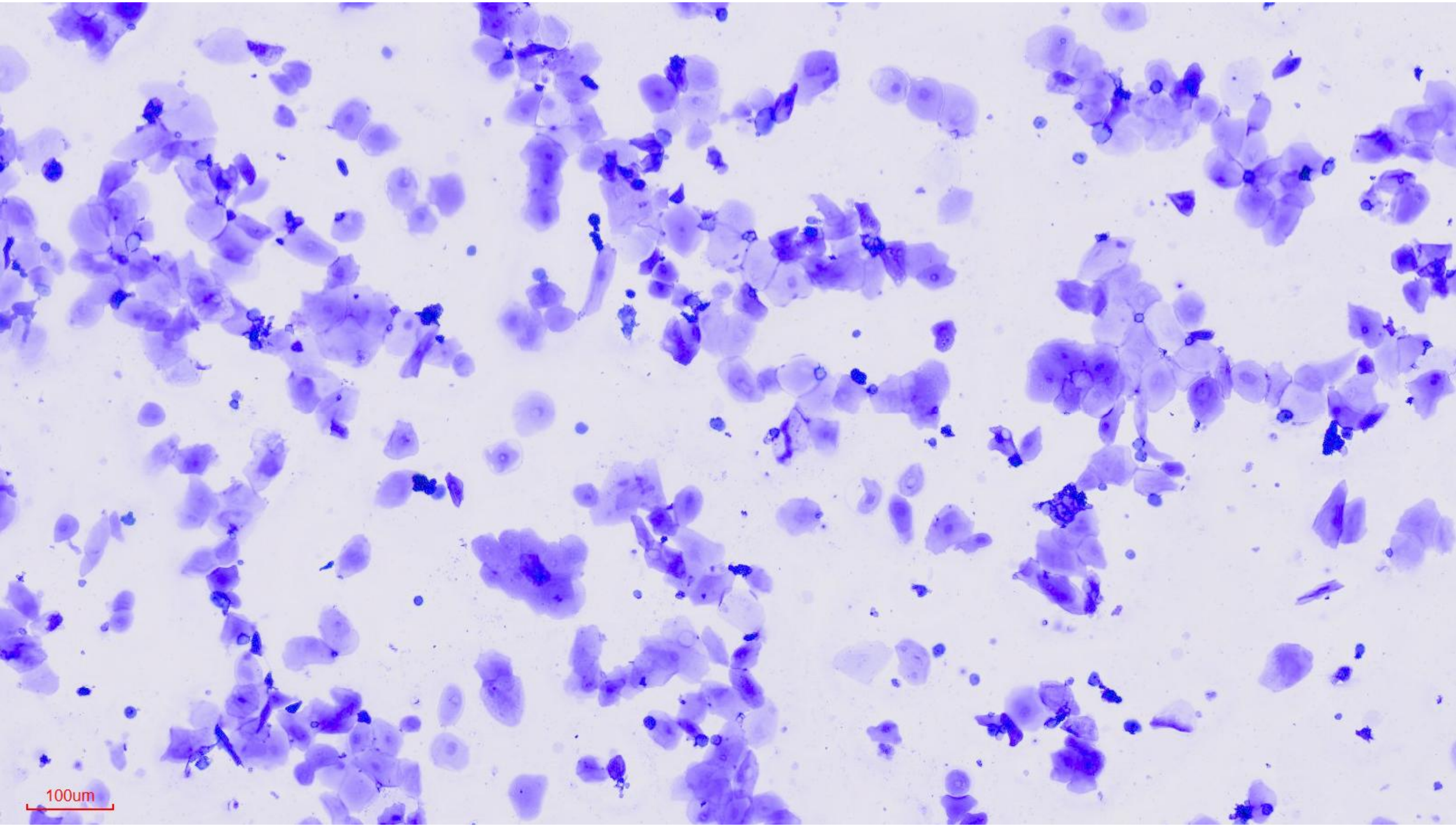

Figure B1 – Ovary
Normal mice

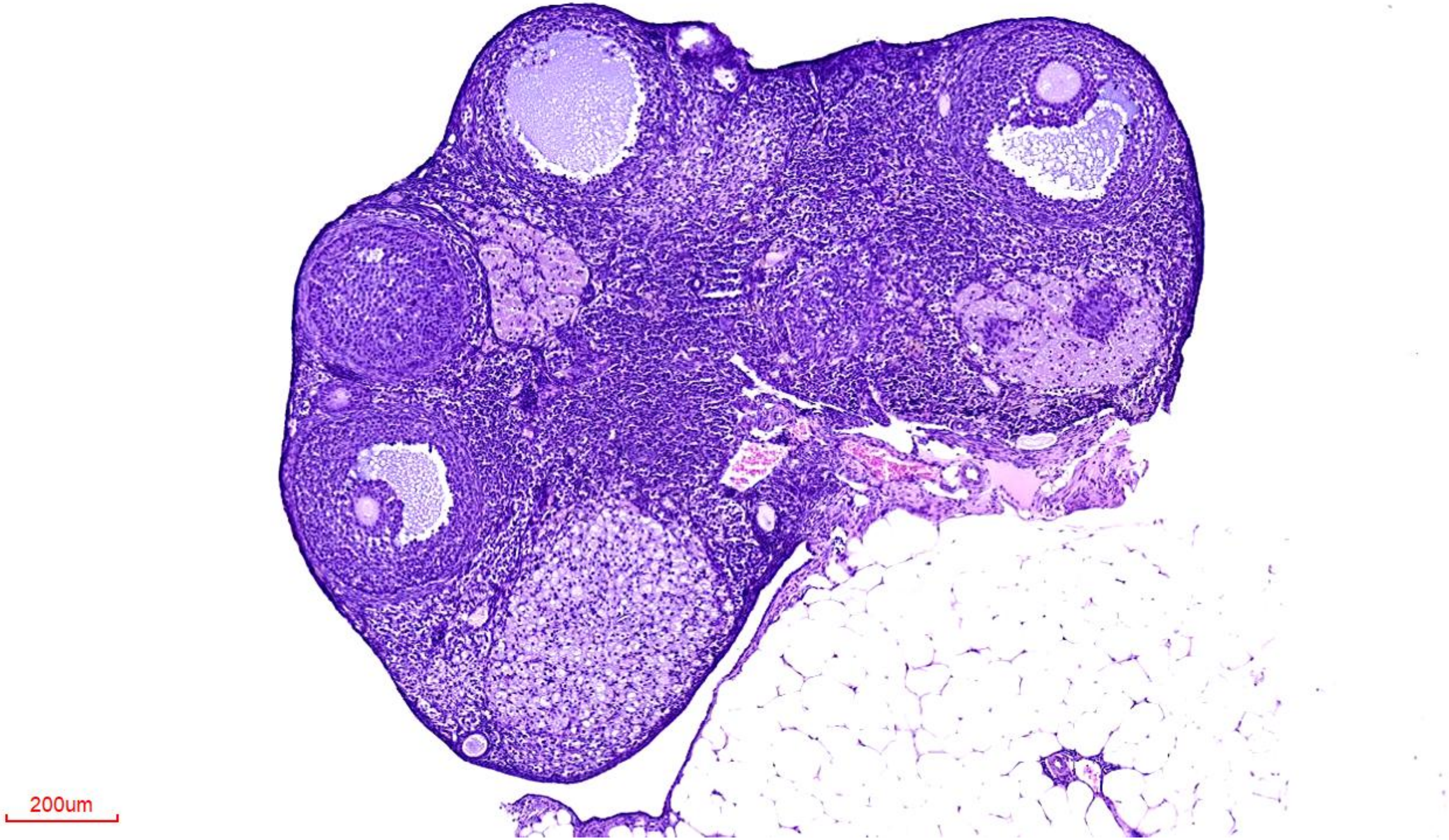

Figure B1 – Ovary
VCD-injected mice

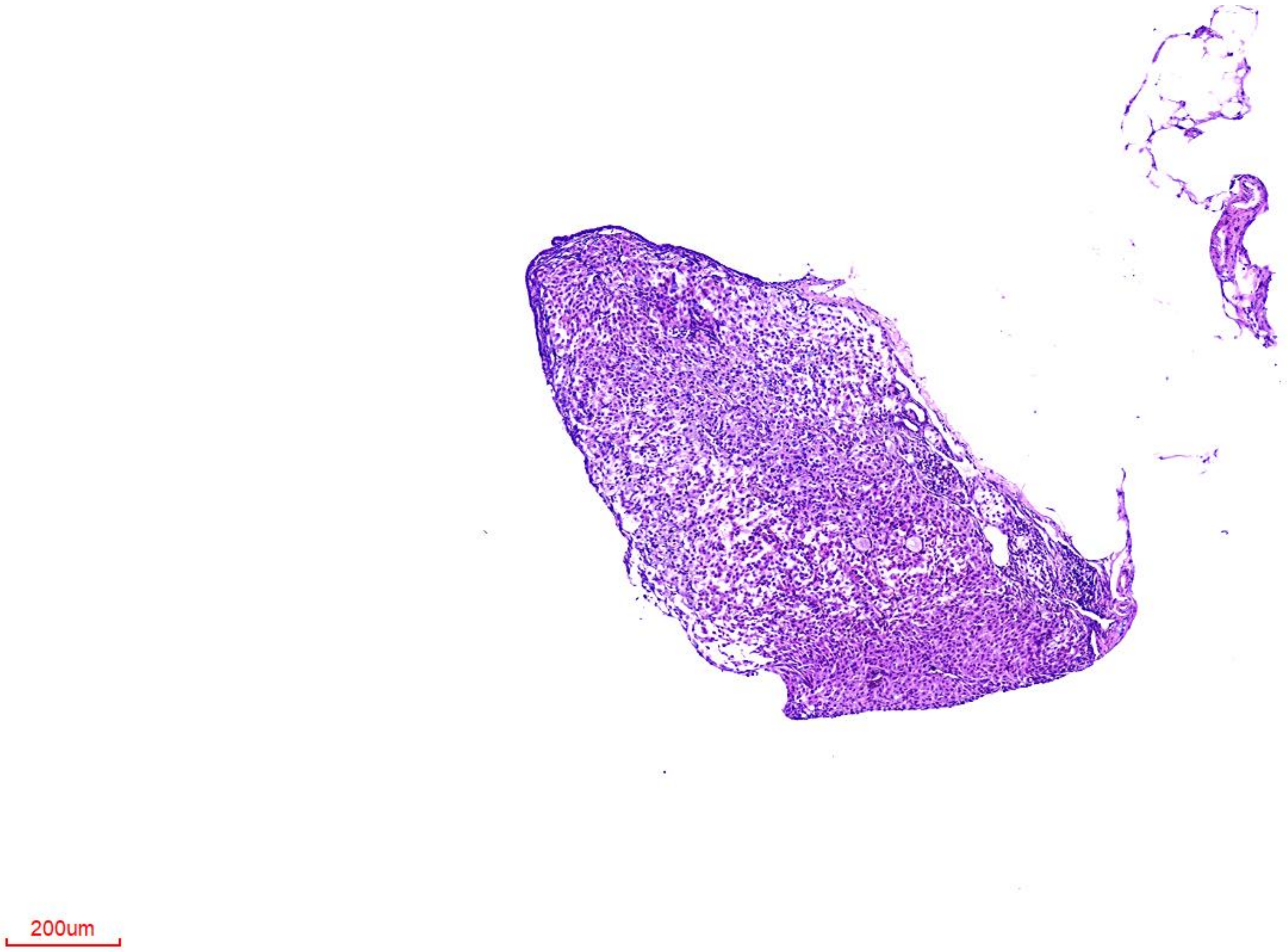

Supplement: Supplementary file 1 [file nutrients-18-03388-s001.zip › File S1, Original microscopic images.pdf]
