# Supplementary material for: Enhanced Estrogenic Activity in Water-Soluble Silybum marianum Complex via Microwave-Assisted Steviol Glycoside-Based Formulation
Source: Nutrients. 2026 Oct 15;18(20):3388. doi: 10.3390/nu18203388 (PMC13708382; doi:10.3390/nu18203388)
Supplement: Supplementary file 1 [file nutrients-18-03388-s001.zip › File S2, Original Western blot images.pdf]

Water-soluble *silybum marianum* complex (*in vitro*)

Figure 5

Figure 5 – p-ERα, ERα

Protein Marker
(SMOBIO, PM2510)

p-ERα

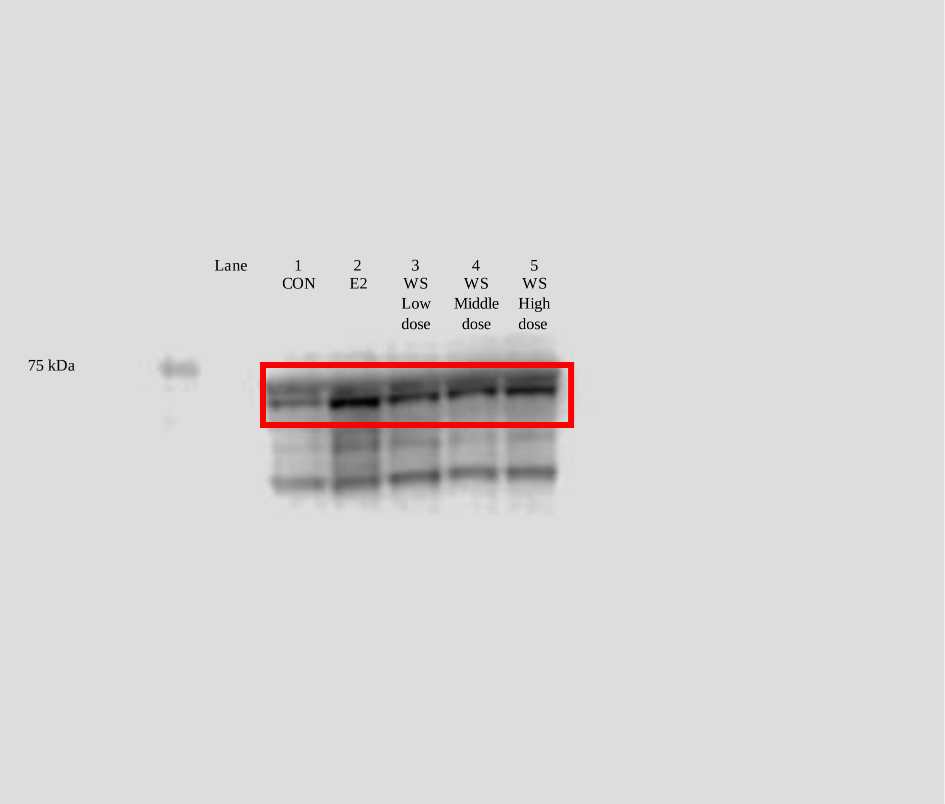

Antibody information

- Phospho-Estrogen Receptor α (ERα)
  - CST, #2511
  - 66 kDa

ERα

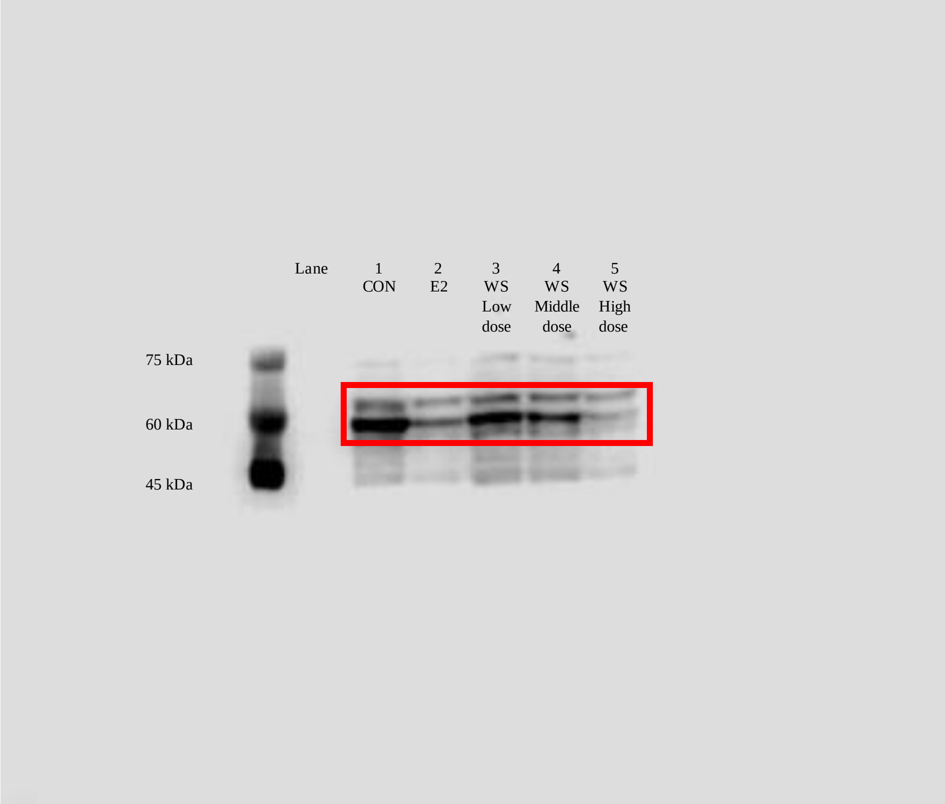

Antibody information

- Estrogen Receptor α (ERα)
  - CST, #8644
  - 66 kDa

Figure 5 – p-ERK, ERK

Protein Marker
(SMOBIO, PM2510)

p-ERK

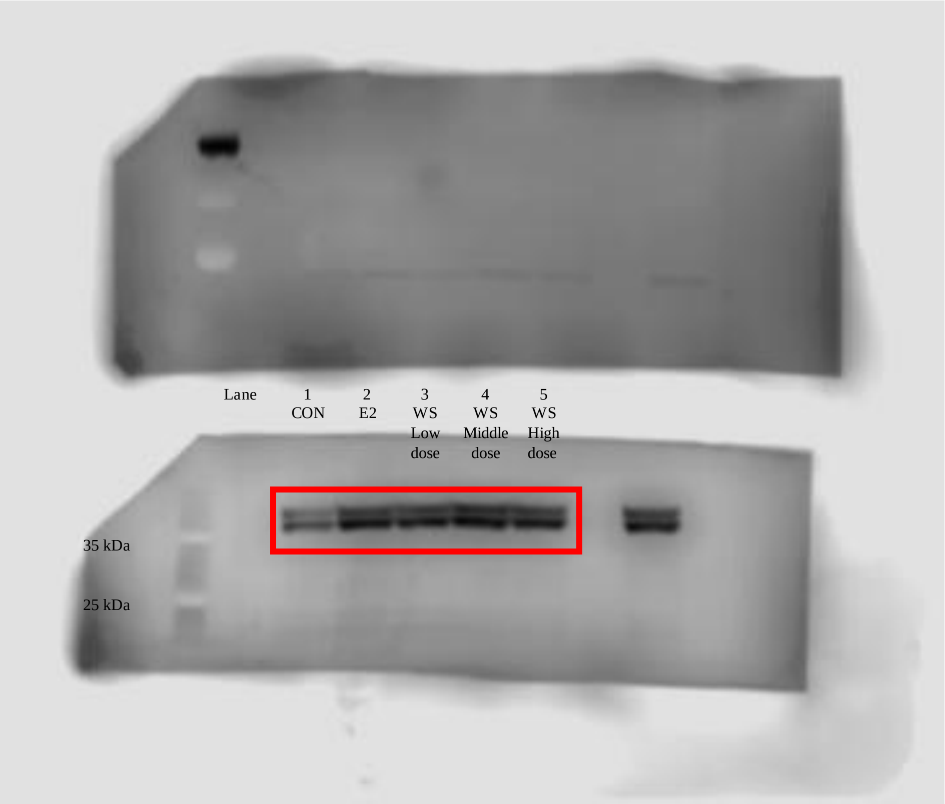

Antibody information

- Phospho-P44/42 MAPK (p-ERK1/2)
  - ➢ CST, #9101
  - ➢ 42,44 kDa

ERK

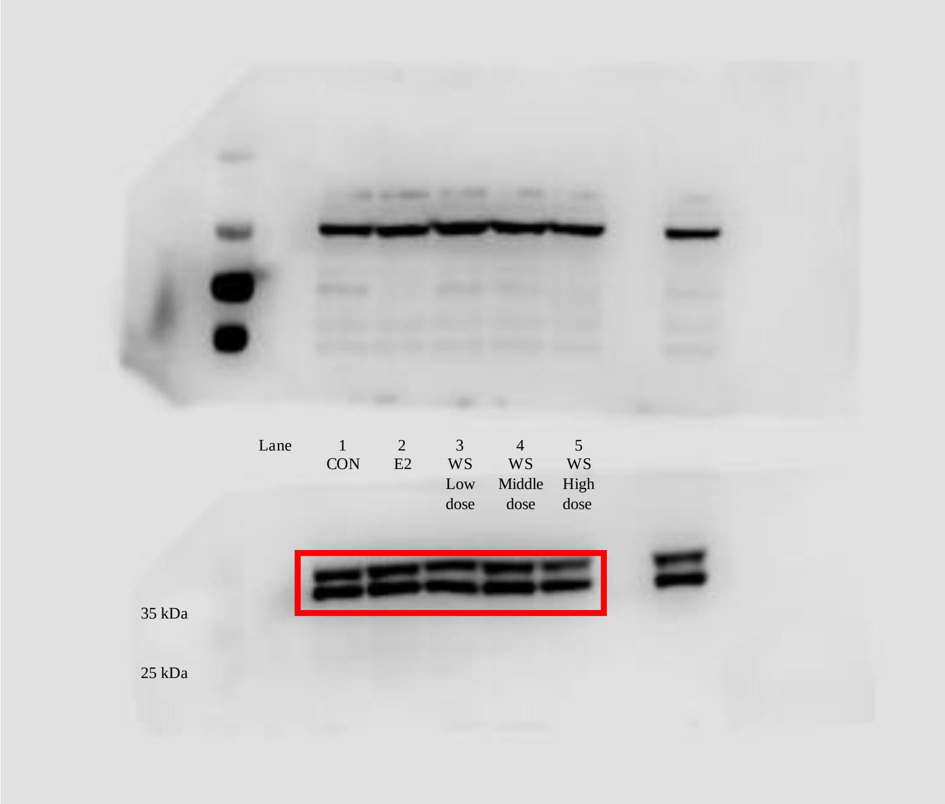

Antibody information

- P44/42 MAPK (ERK1/2)
  - ➢ CST, #9102
  - ➢ 42, 44 kDa

Figure 5 – p-AKT, AKT

Protein Marker
(SMOBIO, PM2510)

p-AKT

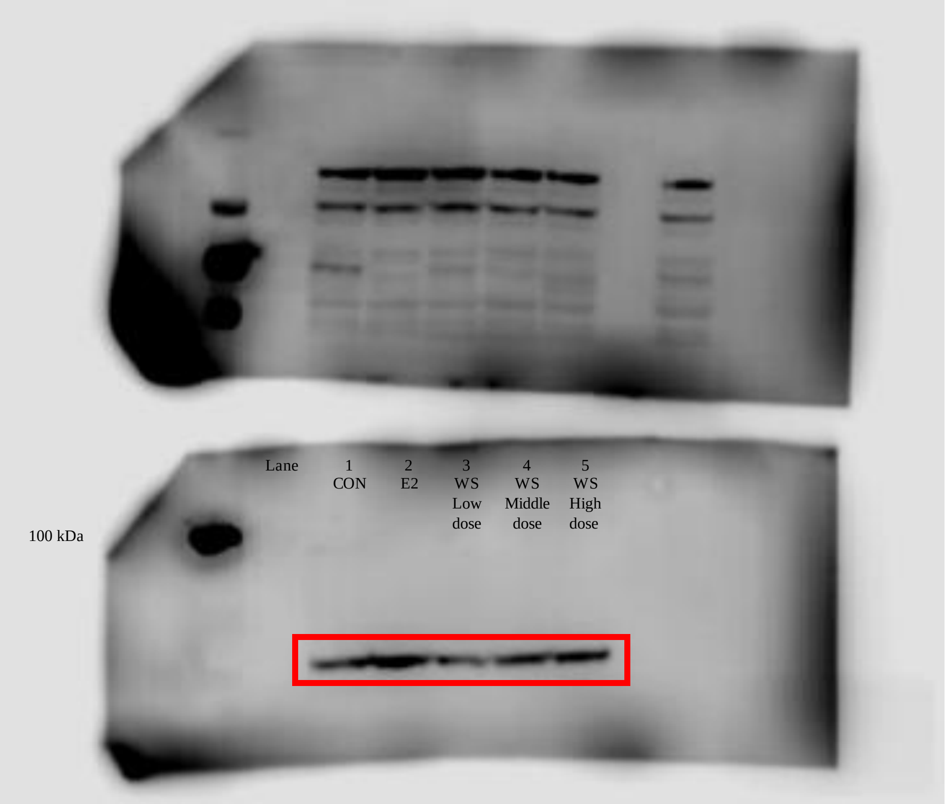

Antibody information

- Phospho-AKT (Ser473)
  - ➢ CST, #4060
  - ➢ 60 kDa

AKT

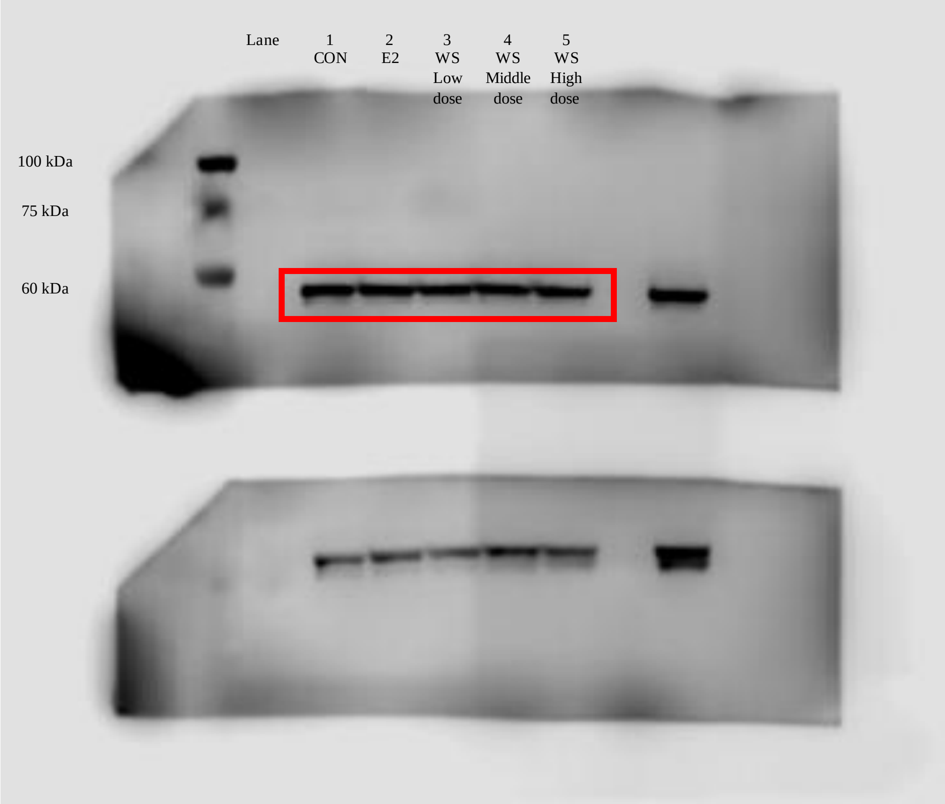

Antibody information

- AKT
  - ➢ CST, #9272
  - ➢ 60 kDa

Figure 5 – β-actin

Protein Marker
(SMOBIO, PM2510)

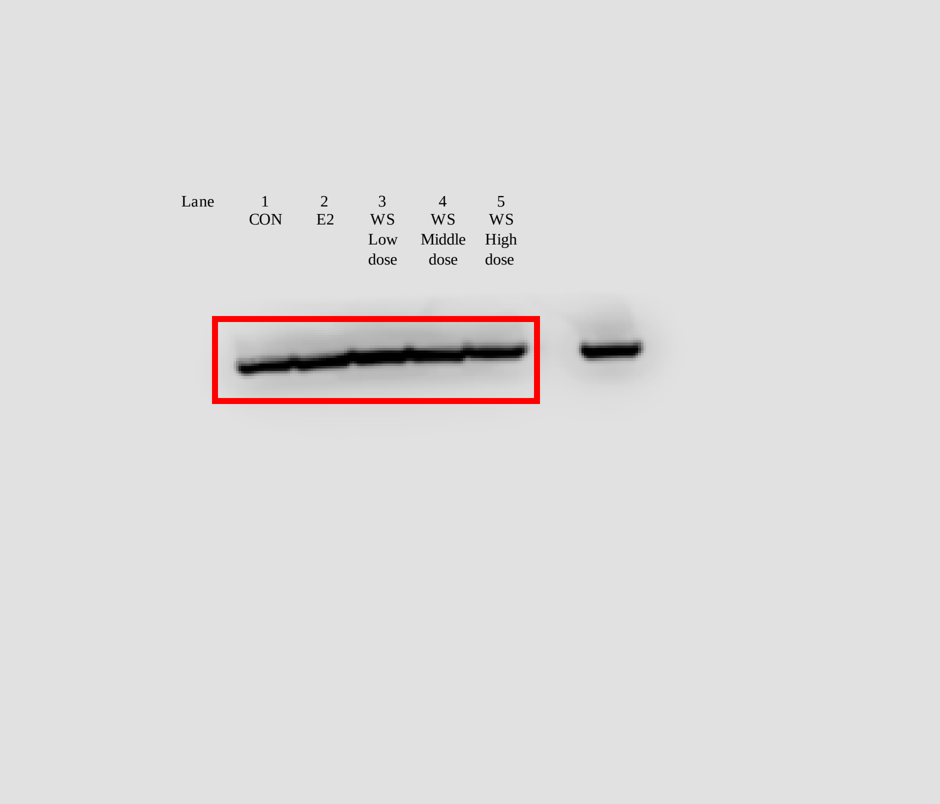

Antibody information

- Beta Actin
  - ➢ Thermo Fisher Scientific, #PA1-183
  - ➢ 42 kDa

Figure 6

Figure 6 – p-ERα, ERα

Protein Marker
(SMOBIO, PM2510)

p-ERα

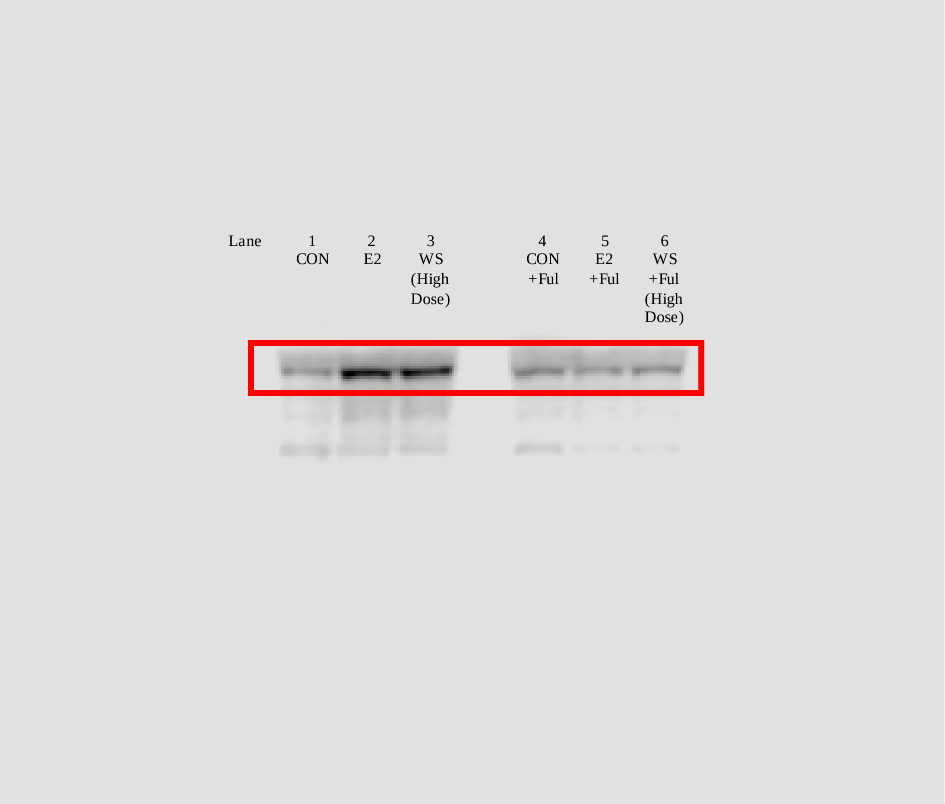

ERα

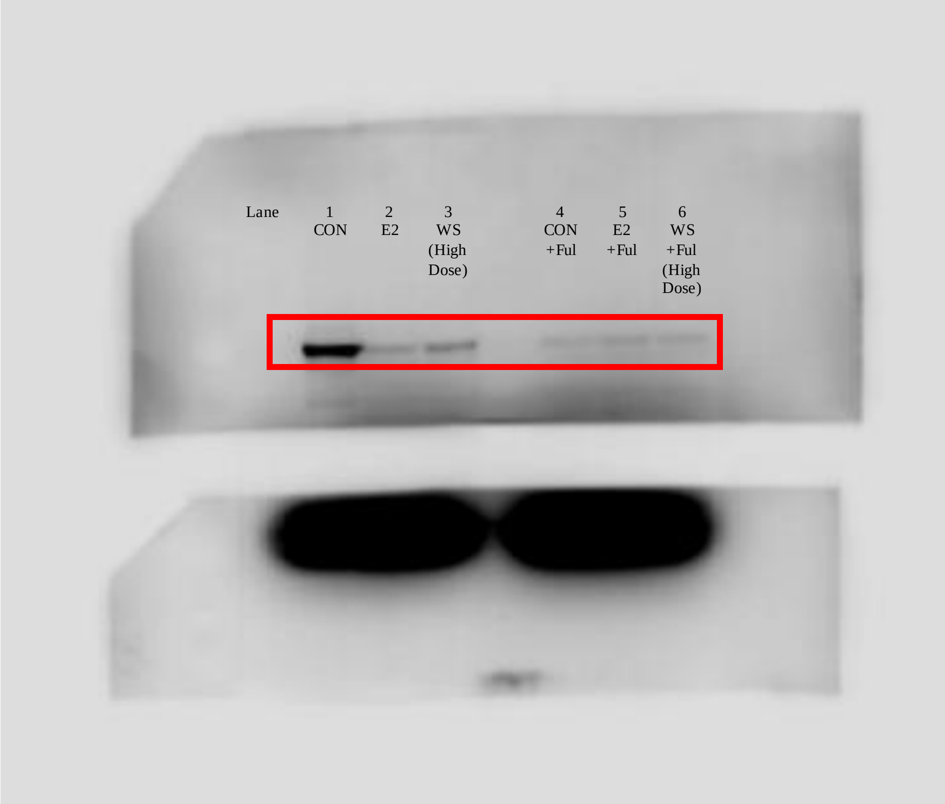

Antibody information

- Phospho-Estrogen Receptor α (ERα)
  - CST, #2511
  - 66 kDa

Antibody information

- Estrogen Receptor α (ERα)
  - CST, #8644
  - 66 kDa

Figure 6 – p-ERK, ERK

Protein Marker
(SMOBIO, PM2510)

p-ERK

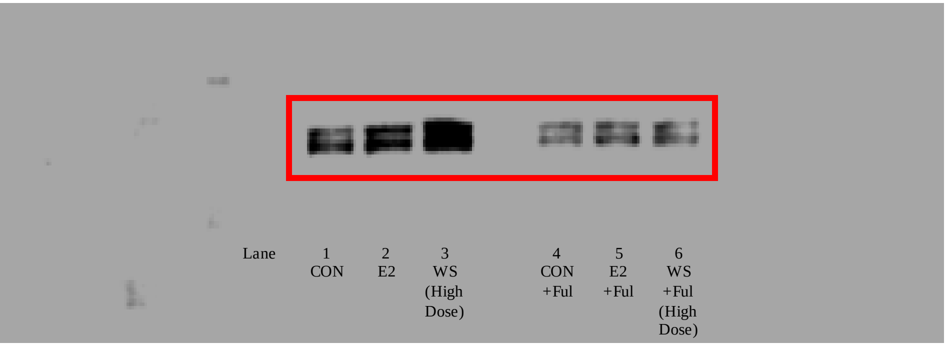

ERK

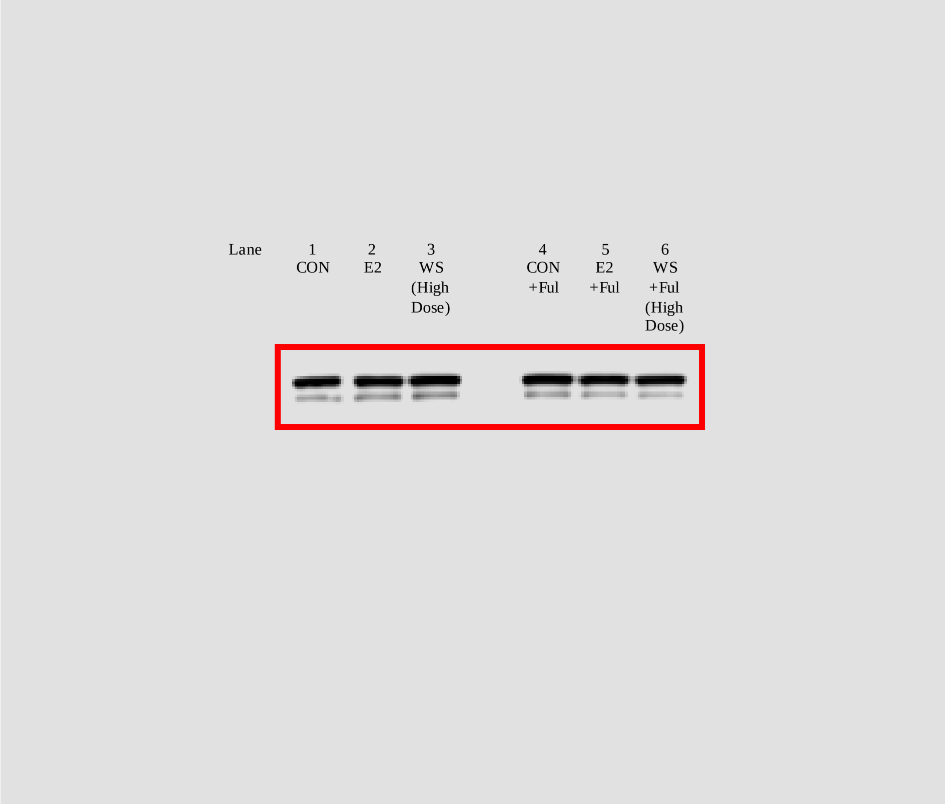

Antibody information

- Phospho-P44/42 MAPK (p-ERK1/2)
  - CST, #9101
  - 42,44 kDa

Antibody information

- P44/42 MAPK (ERK1/2)
  - CST, #9102
  - 42, 44 kDa

Figure 6 – p-AKT, AKT

Protein Marker
(SMOBIO, PM2510)

p-AKT

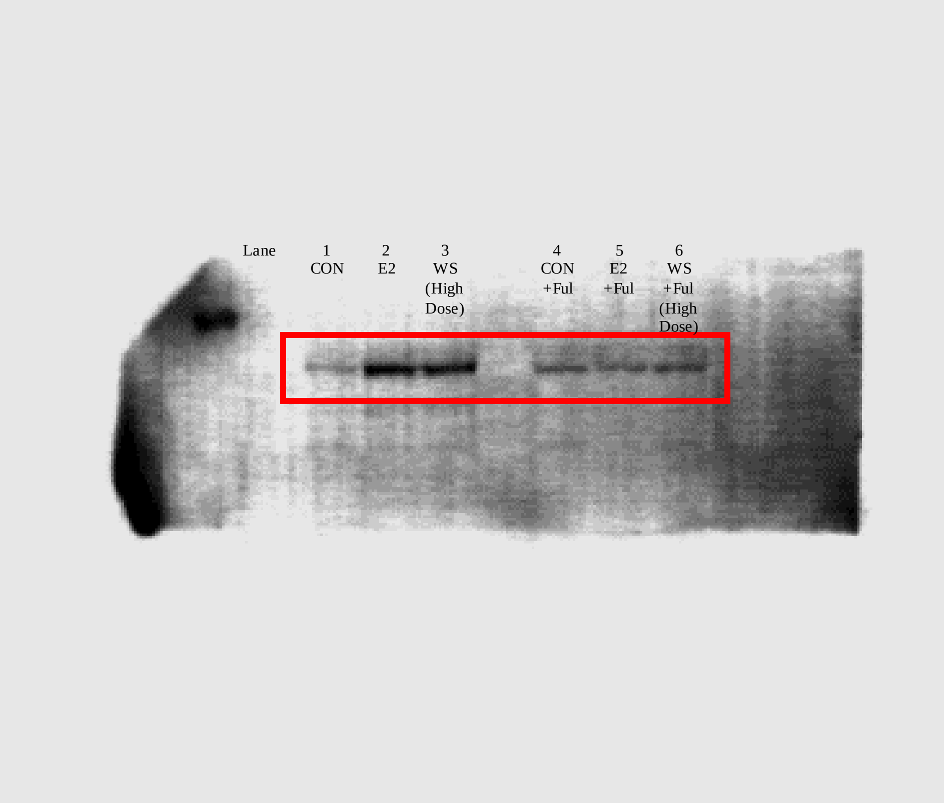

Antibody information

- Phospho-AKT (Ser473)
  - CST, #4060
  - 60 kDa

AKT

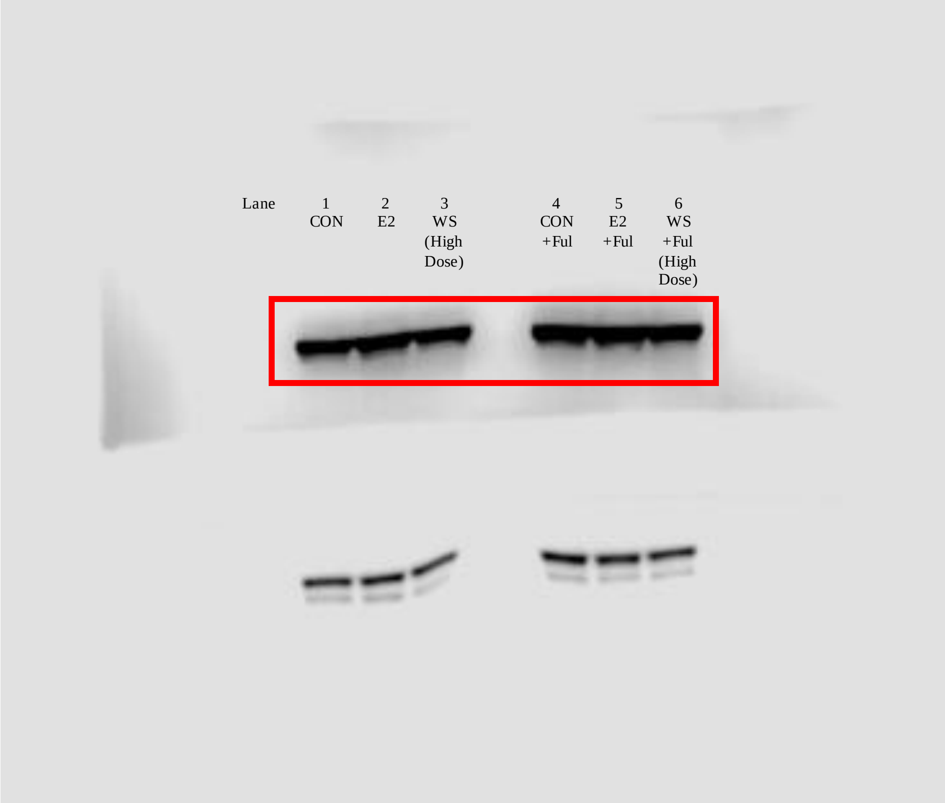

Antibody information

- AKT
  - CST, #9272
  - 60 kDa

Figure 6 – β-actin

Protein Marker
(SMOBIO, PM2510)

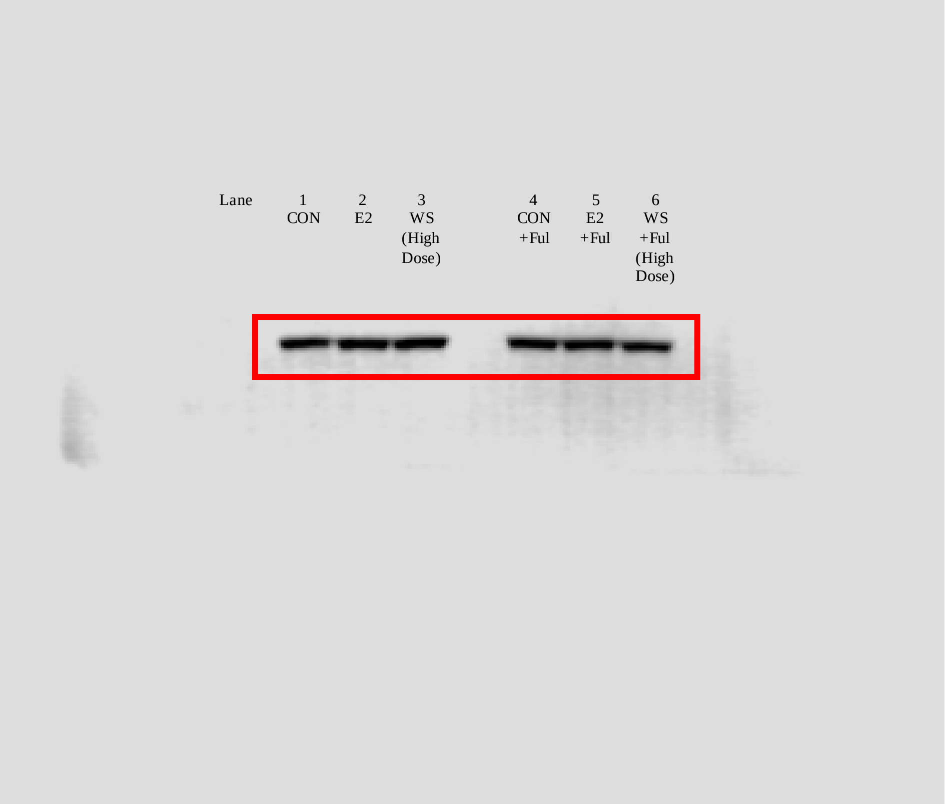

Antibody information

- Beta Actin
  - Thermo Fisher Scientific, #PA1-183
  - 42 kDa
