# Supplementary material for: Enhanced Estrogenic Activity in Water-Soluble Silybum marianum Complex via Microwave-Assisted Steviol Glycoside-Based Formulation
Source: Nutrients. 2026 Oct 15;18(20):3388. doi: 10.3390/nu18203388 (PMC13708382; doi:10.3390/nu18203388)
Supplement: Supplementary file 1 [file nutrients-18-03388-s001.zip › File S3, Statistical analysis results.pdf]

Supplementary Table S1. Post hoc pairwise comparisons for Figure 2

Figure 2A	Comparison	Adjusted p-value	Significance
	CON vs E2	0.00065	***
WS (μg/mL)	CON vs 10	0.2206	ns
	CON vs 25	0.04006	*
	CON vs 50	0.004098	**
	CON vs Fulvestrant	1	ns
	E2 vs E2+Fulvestrant	0.000004	###
WS (μg/mL)	10 vs 10+Fulvestrant	1	ns
	25 vs 25+Fulvestrant	0.2615	#
	50 vs 50+Fulvestrant	0.00319	##
Figure 2B			
	CON vs E2	0.003948	**
	CON vs Genistein	0.003948	**
	CON vs Daidzein	0.003948	**
	CON vs WS	0.003948	**
Figure 2C			
	CON vs E2	0.005351	**
	CON vs WS	0.009023	**
SME (μg/mL)	CON vs 10	1	ns
	CON vs 25	0.3346	ns
	CON vs 50	0.03796	*
SG (μg/mL)	CON vs 10	1	ns
	CON vs 25	1	ns

	CON vs 50	0.772	ns
SME+SG (μg/mL)	CON vs 10	1	ns
	CON vs 25	1	ns
	CON vs 50	0.5547	ns
SME+M (μg/mL)	CON vs 10	1	ns
	CON vs 25	0.5796	ns
	CON vs 50	0.06331	ns
Figure 2D			
	CON vs E2	0.001062	**
WS (μg/mL)	CON vs 10	1	ns
	CON vs 25	0.0229	*
	CON vs 50	0.009185	**
	CON vs Fulvestrant	1	ns
	E2 vs E2+Fulvestrant	0.026	#
WS (μg/mL)	10 vs 10+Fulvestrant	0.04252	#
	25 vs 25+Fulvestrant	0.007499	##
	50 vs 50+Fulvestrant	0.001133	##
Figure 2E			
	CON vs E2	0.009023	**
WS (μg/mL)	CON vs 10	0.02846	*
	CON vs 25	0.02828	*
	CON vs 50	0.01431	*

Supplementary Table S2. Post hoc pairwise comparisons for Figure 5

Figure 4A	Comparison	Adjusted p-value	Significance
	WS vs SME	0.001393	**
	WS vs SG	0.002064	##
Figure 4B			
	WS vs SME	0.01092	*
	WS vs SG	0.001593	##

Supplementary Table S3. Post hoc pairwise comparisons for Figure 6

Figure 5B	Comparison	Adjusted p-value	Significance
	CON vs E2	0.004959	**
WS (μg/mL)	CON vs 10	1	ns
	CON vs 25	0.002376	**
	CON vs 50	0.003401	**
Figure 5C			
	CON vs E2	0.004959	**
WS (μg/mL)	CON vs 10	1	ns
	CON vs 25	0.002376	**
	CON vs 50	0.003401	**
Figure 5D			
	CON vs E2	0.00006781	***
WS (μg/mL)	CON vs 10	1	ns
	CON vs 25	0.0126	*
	CON vs 50	0.005017	**

Supplementary Table S4. Post hoc pairwise comparisons for Figure 7

Figure 6B	Comparison	Adjusted p-value	Significance
	CON vs Fulvestrant	0.4834	ns
	E2 vs E2+Fulvestrant	0.02066	*
	WS vs WS+Fulvestrant	0.00966	**
Figure 6C			
	CON vs Fulvestrant	0.531	ns
	E2 vs E2+Fulvestrant	0.00953	**
	WS vs WS+Fulvestrant	0.01533	*

Supplementary Table S5. Post hoc pairwise comparisons for Figure 8

Figure 5B	Comparison	Adjusted p-value	Significance
WS (mg/kg/day)	NC vs MC	0.009387	##
	MC vs PC	0.0003167	***
	MC vs 10	1	ns
	MC vs 25	0.01681	*
	MC vs 50	0.008164	**
Figure 5C			
WS (mg/kg/day)	NC vs MC	0.0002596	###
	MC vs PC	0.00009261	***
	MC vs 10	1	ns
	MC vs 25	0.006403	**
	MC vs 50	0.0004071	***
Figure 5D			
WS (mg/kg/day)	NC vs MC	0.02092	#
	MC vs PC	0.0009297	***
	MC vs 10	1	ns
	MC vs 25	0.3034	ns
	MC vs 50	0.004408	**
